# Epigenetic Alterations Affecting Transcription Factors and Signaling Pathways in Stromal Cells of Endometriosis

**DOI:** 10.1371/journal.pone.0170859

**Published:** 2017-01-26

**Authors:** Iveta Yotova, Emily Hsu, Catherine Do, Aulona Gaba, Matthias Sczabolcs, Sabine Dekan, Lukas Kenner, Rene Wenzl, Benjamin Tycko

**Affiliations:** 1 Institute for Cancer Genetics, Herbert Irving Comprehensive Cancer Center, Columbia University Medical Center, New York, New York, United States of America; 2 Department of Gynecology and Gynecological Oncology, University Clinic of Obstetrics and Gynecology, Medical University of Vienna, Vienna, Austria; 3 Department of Pathology and Cell Biology, Herbert Irving Comprehensive Cancer Center, Columbia University Medical Center, New York, New York, United States of America; 4 Department of Experimental Pathology, Clinical Institute of Pathology, University Clinic of Obstetrics and Gynecology, Medical University of Vienna, Vienna, Austria; 5 Pathology Laboratory Animal Pathology University of Veterinary Medicine Vienna, Vienna, Austria; 6 Ludwig Boltzmann Institute for Cancer Research, Vienna, Austria; New York University School of Medicine, UNITED STATES

## Abstract

Endometriosis is characterized by growth of endometrial-like tissue outside the uterine cavity. Since its pathogenesis may involve epigenetic changes, we used Illumina 450K Methylation Beadchips to profile CpG methylation in endometriosis stromal cells compared to stromal cells from normal endometrium. We validated and extended the Beadchip data using bisulfite sequencing (bis-seq), and analyzed differential methylation (DM) at the CpG-level and by an element-level classification for groups of CpGs in chromatin domains. Genes found to have DM included examples encoding transporters (*SLC22A23*), signaling components (*BDNF*, *DAPK1*, *ROR1*, and *WNT5A*) and transcription factors (*GATA* family, *HAND2*, *HOXA* cluster, *NR5A1*, *OSR2*, *TBX3*). Intriguingly, among the TF genes with DM we also found *JAZF1*, a proto-oncogene affected by chromosomal translocations in endometrial stromal tumors. Using RNA-Seq we identified a subset of the DM genes showing differential expression (DE), with the likelihood of DE increasing with the extent of the DM and its location in enhancer elements. Supporting functional relevance, treatment of stromal cells with the hypomethylating drug 5aza-dC led to activation of *DAPK1* and *SLC22A23* and repression of *HAND2*, *JAZF1*, *OSR2*, and *ROR1* mRNA expression. We found that global 5hmC is decreased in endometriotic versus normal epithelial but not stroma cells, and for *JAZF1* and *BDNF* examined by oxidative bis-seq, found that when 5hmC is detected, patterns of 5hmC paralleled those of 5mC. Together with prior studies, these results define a consistent epigenetic signature in endometriosis stromal cells and nominate specific transcriptional and signaling pathways as therapeutic targets.

## Introduction

Endometriosis is characterized by growth of endometrial-like tissue outside the uterine cavity. As a hormone-driven disorder it affects women of reproductive age, and it is associated with chronic pelvic pain, pelvic inflammatory reactions and infertility. Although it is not a malignant condition, it shares its metastasizing-like biological behavior and certain aspects of gene expression with cancers [1]. In healthy individuals, the development and the maintenance of the decidua is dependent on progesterone, and a hormonal withdrawal in the absence of pregnancy provokes apoptosis and shedding of the endometrium and differentiated decidual cells during menstruation [2]; this physiological response is altered in women with endometriosis partly due to progesterone-resistance of the ectopic endometrial tissue [3].

Multiple predisposing factors of genetic, epigenetic and environmental origin, combined with an altered immune response, are thought to contribute to survival of endometrial cells outside the uterine cavity in the endometriotic lesions [4]. Since there are important but to date only partly characterized interactions between epithelial cells, inflammatory cells with their associated cytokines, and mesenchymal stromal cells in these lesions (e.g. [5–7]), a full elucidation of the pathogenic mechanisms will require testing multiple biological hypotheses. Among these possibilities, epigenetic changes in endometriosis have come under scrutiny. Initial reports focused on DNA methylation changes in candidate genes associated with sex-steroid hormone signaling and the dysregulation of endometrial decidualization [8]: losses of methylation in gene promoters for aromatase [9], steroidogenic factor-1 [10] and estrogen receptor beta [11] were associated with local estrogen production and enhanced estrogen signaling in ectopic whole endometriotic tissue compared to control uterine endometrium. Hypermethylation of promoter regions of genes involved in implantation including those encoding the progesterone receptor, homeobox A10, and e-cadherin were reported in endometrium of patients with endometriosis (reviewed in: [8]) and several other genes have also been reported to show abnormal CpG methylation in endometriotic lesions [12]. Recently, altered promoter methylation in eutopic endometrial cells was suggested as a possible mechanism in women who will develop endometriosis later in life [13].

In addition to these candidate gene studies, methods for genome-wide profiling of differential methylation (DM) have advanced quickly, and studies by us and many others using microarrays such as 450K Illumina Methylation Beadchips, and massively parallel bisulfite sequencing (bis-seq), have shown that not only promoter regions but also intragenic, intergenic and enhancer sequences have dynamic DNA methylation patterns in cell differentiation and disease [14, 15]. Methylation arrays have been used by six independent groups to study endometriosis, with four reports comparing DNA methylation patterns in whole tissue samples of patients with endometriosis versus healthy controls [16–19] and two other studies reporting on cultured stromal cells from control endometrium and endometriosis [20, 21]. Here we use 450K Methylation Beadchips, with extensive validations by bis-seq, and with parallel genome wide expression profiling by RNA-Seq, to compare epigenetic patterning in endometriosis stromal cells at ovarian ectopic sites (OESC) vs. control endometrial stromal cells (CESC). Our findings confirm some of the results from prior investigations and highlight additional examples of DM genes that point to targetable biological pathways for future therapies of endometriosis. In addition, we present a useful method for analyzing DM at the level of chromatin elements, and we uncover mechanistically informative relationships between DM and differential expression (DE) that may be relevant not only to endometriosis but also to other human disorders.

## Materials and Methods

### Tissue samples

All samples used for analysis in this study were obtained from premenopausal women undergoing laparoscopic surgery because of suspected endometriosis, pelvic pain of unknown origin, adnexal cysts, infertility work-up or leiomyoma uteri. Patients with history of any malignant disease, acute inflammatory process, infection, or systemic autoimmune disorders were excluded from study participation. The presence or absence of endometriosis was confirmed visually by laparoscopy and additional histopathological analysis. The study was approved by the institutional ethics committee of the Medical University of Vienna (EK 545/2010). All patients gave their verbal and written informed consent prior to study inclusion.

### Stromal cell cultures

The primary tissue samples and OESC and CESC cells analyzed in this study are in **S1 Table**. Cryopreserved endometrial tissue obtained by diagnostic laparoscopy was minced and incubated with collagenase (Sigma-Aldrich, St. Louis, MO) at 37°C for 10 min., followed by filtration, as previously described [22]. This method produces 95–99% pure stromal cells. The purity of these stromal cells at passage one was evaluated by immunofluorescence analysis using antibodies against vimentin (stromal cell marker), cytokeratin7 (epithelial cell marker) and CD45 (leukocyte marker), which showed that all cultures were 98–99% pure stromal mesenchymal cells. The cells were then cultured as previously described [23]. Briefly, the cells were cultured on fibronectin-collagen (Gibco, Grand Island, NY) coated dishes in DMEM-F12 without phenol red (Gibco) supplemented with 10% fetal bovine serum (FBS) (Gibco), 2 mM L-glutamine (Gibco) and 1% antibiotics–antimycotic (Gibco) up to passage 3. To exclude influences of the serum derived steroid hormones on DNA methylation and expression in the cultures, the cells were grown in culture medium containing 10% charcoal stripped fetal bovine serum (CS-FBS; Gibco).

### Immunohistochemistry (IHC) and immunofluorescence (IF) analyses of archival tissue samples

Paraffin embedded tissue samples from controls (n = 14) and endometriosis cases (n = 14) collected in the Pathology Department of the Medical University of Vienna were used for immunohistochemical (IHC) staining of WNT5A. Six tissue samples (3 controls and 3 endometriosis) from the Pathology Department of Columbia University Medical Center were used for 5mC and 5hmC analysis by immunofluorescence (IF). For IHC, antigen retrieval was performed by autoclaving the slides in 10 mM Sodium citrate buffer using DAKO target retrieval solution pH = 6 (DAKO, Carpinteria, CA) for 20 min. The slides were further blocked with 3% sodium peroxide (Gatt-KOLLER, Absam, Austria) for 10 min, stained with avidin (10 min), biotin (10 min) and again blocked for 7 min using Superstain Horseradish Peroxidase (HPR) system (Empire Genomics, Buffalo, NY, USA; IDST 1007). An anti-WNT5A-antibody (Cell Signaling Technology, Danvers, MA) at 1:1000 dilution was applied for identification of WNT5A protein, with incubation of the slides overnight at 4°C. Amino ethyl carbazole (AEC) development (Labs Biotechnology, American Fork, UT) was performed for 2 min. For double immunofluorescence (IF) of 5mC and 5hmC, we detected 5mC using a mouse monoclonal antibody (Ab-1; Calbiochem, San Diego, CA), and we used a polyclonal antibody (anti-5hmC, Active Motif, CA, USA) to detect 5hmC, as previously described [24].

### CpG methylation profiling using Illumina 450K Methylation Beadchips

The amount and integrity of genomic DNA was assessed by agarose gel electrophoresis with ethidium bromide staining and by PicoGreen® dsDNA quantitation assays (Life Technologies, Carlsbad, CA, USA). Genomic DNA, 500 ng, was bisulfite converted and analyzed per the manufacturer’s instructions for Illumina HumanMethylation450K Beadchips, with all assays performed at the Roswell Park Cancer Institute Genomics Shared Resource, New York, USA. The BeadChip-based methylation assays entail bisulfite conversion of the genomic DNA followed by primer extensions to query the percent methylation at each of 485,000 (450K) CpG dinucleotides, covering sequences in and around promoter-associated and non-promoter-associated CpG-islands (CGIs), as well as many non-island promoter regions, associated with 99% of RefSeq genes. Data were processed using Genome Studio software, which calculates the percent methylation (AVG_Beta) at each CpG queried by the array, after background correction and normalization to internal controls.

### Analysis of the 450K BeadChip data at the individual CpG and regulatory element levels

As initial data cleaning, AVG_Beta values with detection p-values>.05 were designated as missing values. All probes mapping to the X or Y chromosome were removed, as were poorly performing probes with missing values in more than 20% of the samples and probes querying a common SNP (DbSNP137, minor allele frequency>0.01). After these steps, 452,704 probes (queried CpGs) remained for analysis. In addition to performing a standard analysis for DM at each individual CpG using p-value (with FDR) and average difference criteria, we additionally performed a modified element-level analysis, grouping together contiguous CpGs in promoter, enhancer and insulator sequences. Chromatin state data from ENCODE and related projects [25] were downloaded from the USCS browser and analyzed in a non-cell specific manner. Multiple CpGs mapping the same regulatory sequence and distant from less than 500 bp were grouped into the same segment. A 500 bp window was defined around single CpGs mapping a given regulatory element and large segments (>500 bp) were tiled into 500 bp segments including the 100 bp upstream and downstream flanking regions and with a 100 bp overlap between tiles. Fractional methylation values were then average across each 500 bp window. P-values for the case/control comparison were calculated using Student’s T-tests and false discovery rates were calculated using the Benjamini-Hochberg method. For the CpG-level, we defined DM CpGs using stringent criteria, FDR<0.05 and absolute difference in fractional methylation >0.15, as well as more lenient criteria, nominal p-value<0.05 and at least 2 CpGs in each gene with p-value<0.05 and fractional methylation >0.15. Similarly, DM segments were defined using stringent criteria, FDR<0.05 and absolute difference in averaged fractional methylation >0.10 (or >0.15 when the segment contained a single CpG), as well as more lenient criteria, nominal p-value<0.05 and at least 2 segments in each gene with p-value<0.05 and fractional methylation >0.10 (or >0.15 for single CpG segment). Analyses were performed using R and STATA statistical software.

### Gene set enrichment analysis (GSEA) and gene ontology enrichment analysis (GOEA) of DM and DE loci and test for correlations of DM with chromatin states

To test whether the DM genes, identified by our stringent criteria, are associated with specific biological function we performed gene ontology enrichment analysis using DAVID (https://david.ncifcrf.gov/) [26], [27] and GSEA-Broad Institute (www.broadinstitute.org/gsea) [28], [29] software. In the DAVID annotation system, Fisher’s Exact test is adopted to measure the gene-enrichment in annotation terms. Gene ontology (GO) terms showing a Fisher’s Exact p-value <0.05 were considered significantly enriched among DM genes. In GSEA we used the Molecular Signature Database (MSigDB) to investigate the overlap between our gene lists and known annotated gene sets. Gene sets showing FDR q-value <0. 005 were considered as significantly enriched among DM genes. We considered the biological processes associated with significantly enriched GO terms or MSigDB gene sets as potentially relevant for endometriosis. Chromatin state data from ENCODE [25] were analyzed in a non-cell type-specific manner: we focused on chromatin states associated with promoter (active, weak or poised), enhancer (active or poised) or insulator in at least one queried cell line. To test whether DM CpGs occur at specific chromatin states more often than random expectation, we used univariate logistic regressions with the presence or absence of DM as the dependent variable and the tested sequence feature as the explanatory covariate, as described in our recent work [15, 30]. Enrichment of a given state among DM CpGs was estimated by the odd ratios (ORs) and log_2_(OR) was used to visualize under-representation and enrichment in a symmetrical manner.

### Standard and oxidative bis-seq

Genomic DNA, 500 ng, was bisulfite-converted using the EpiTect Bisulfite Kit (Qiagen, CA, USA). Sequences spanning the DM CpGs were amplified by PCR, using primers designed in MethPrimer [31], the products cloned in bacteria (TopoTA Cloning Kit; Thermo-Fisher Scientific, MA, USA) and multiple clones sequenced. We evaluated the relative contributions of 5mC and 5hmC to DM at selected loci using the TrueMethyl^TM^6 kit (CEGX, Cambridge, UK). This oxidative chemical conversion-based approach uses oxidative bis-seq of multiple clones to score 5mC-only while standard bis-seq is used in parallel to score 5mC+5hmC, so that the percent contribution of 5hmC to net methylation at each CpG can be inferred from the difference between oxidative and standard bisulfite conversion. For both standard and oxidative bis-seq at least 10 independent clones were sequenced per amplicon per DNA sample. Primer sequences are in **S7 Table.**

### RNA-seq and analysis of correlations between DM and DE

RNA was isolated using TRIZOL reagent (Invitrogen, MA, USA) and RNA integrity was confirmed as RIN>7 on a Bio Analyzer (Agilent Technologies, CA, USA). Poly-A pull-down was used to enrich for mRNAs, and libraries were prepared using the Illumina TruSeq RNA kit. Libraries were pooled and sequenced on an Illumina HiSeq2000 machine with 100 bp paired-end reads. RTA (Illumina, San Diego, CA, USA) was used for base calling and bcl2fastq (version 1.8.4) for converting BCL to FASTQ format, coupled with adaptor trimming. The reads were mapped to the human reference genome (NCBI/build37.2) using Tophat [32] (version 2.0.4) with 4 mismatches (—read-mismatches = 4) and 10 maximum multiple hits (—max-multihits = 10). The relative expression level of genes was estimate by FPKM (Fragments Per Kilobase of transcript per Million mapped reads) using cufflinks [33] (version 2.0.2) with default settings. Genes with very low levels of expression, defined by average FPKM values < 1 in both OESC and CESC groups were excluded from further analysis. To identify DM loci for which CpG methylation correlates with expression, we regressed, for each expressed gene queried by RNA-seq and Illumina 450K Beadchips, its FPKM value against the fractional methylation of each CpG or groups of CpGs in each sequence element within and flanking the gene (within 1.5 kb upstream of the gene transcription starting site). Significant correlation was defined as p<0.005 and rho correlation coefficient >0.7 (corresponding to a R^2^>0.5). We then superimposed the lenient DM CpG- and segment-level lists with the list of CpGs/segments with correlation between expression and methylation.

### Treatment of cells with 5aza-dC

Equal numbers of OESC and CESC cells (passage 3) were seeded in 10 cm dishes. After the cells reached about 50% confluency, 5aza-dC was added to a final concentration of 0, 1.0, 1.5 or 2.0 micromolar. The culture medium was changed every 24 hours, with addition of freshly prepared 5aza-dC. These concentrations of the drug affected cell proliferation but did not cause noticeable cell death: changes in the proliferation rate of the cells were noticeable after the first 24 hours of treatment and became more pronounced after 72 hours, when the cells were harvested for RNA isolation. This treatment was performed in biological and technical replicates for the OESC and CESC. RNA isolation, followed by DNase I treatment (Ambion, MA, USA) was performed as described above and the total RNA was further used for reverse transcription and Q-PCR analysis of *JAZF1*, *ROR1*, *SLC22A23*, *HAND2*, *HAND2as*, *DAPK1* and *WNT5A* genes.

### Quantitative PCR (Q-PCR) for measuring mRNA expression

Total RNA was reverse transcribed with SuperScript® III First-Strand Synthesis Reverse Transcriptase (Life Sciences Advance Technology, St Petersburg, FL), with priming using a mixture of oligo-d (T) and random hexamers. Q-PCR was performed in triplicate in 96-well optical plates and repeated two or three times using independent cDNA sets, all of which gave consistent results. Each reaction contained 1X Power SYBR Green PCR master mix (Applied Biosystems, MA, USA) and 0.2 μM of each specific primer pair, which were designed using online Real Time PCR tool (IDT). Q-PCR was performed using a 7500 Fast Real-Time PCR System (Applied Biosystems), or a StepOnePlus instrument (Bio-Rad, CA, USA), with an initial denaturation for 10 min at 95°C, primer annealing at 50°C for 2 min, followed by 40 cycles of 15 secs at 95°C and 1 min at 60°C. The relative expression of target genes was calculated by the delta-CT method as described [34], with normalization using either *B2MG* or *CSNK1D* housekeeping genes. The average Ct values were ≤30 except for JAZF1as and ROR1 transcripts showing average Ct-value of 32 cycles (for ROR1 in CESC and for JAZF1as in both cell types), for each of the assayed genes using 2-fold dilutions of the SuperScript-generated cDNA preparations. The Q-PCR primer sequences are in **S8 Table**. Q-PCR for the *WNT5A* gene was performed using TaqMan primers (Applied Biosystems) and probes for WNT5A (Applied Biosystems, Hs00998537_m1) and the TBP house-keeping gene, with ROX reference dye (Thermo Fisher Scientific) and InnuMIX Q-PCR Mastermix (Analytik Jena, Jena, Germany).

### Quantitation of secreted BDNF by ELISA

The levels of secreted BDNF in the supernatants of cultured OESC and CESC were measured using the Biosensis human BDNF Rapid ELISA Kit (Biosensis, Thebarton, Australia; BEK-2211-1P) following the manufacturer’s protocol. Cells were grown in DMEM-F12 media without phenol red (Gibco) supplemented with 0.5% charcoal stripped-FBS (CS-FBS; Gibco), 2 mM L-glutamine (Gibco) and 1% antibiotics–antimycotic (Gibco) for four days. The supernatants were collected, particles were removed by centrifugation (10,000 x g for 5 minutes), and the supernatants were diluted 1:4 and further subjected to enzyme-linked immunosorbent assay (ELISA). A total of 6 independent samples per group, with technical triplicates, were analyzed and the levels of secreted BDNF were calculated in pg/ml.

## Results

### Differential CpG methylation in endometriosis stromal cells vs. stromal cells from normal endometrium

**Fig 1:**
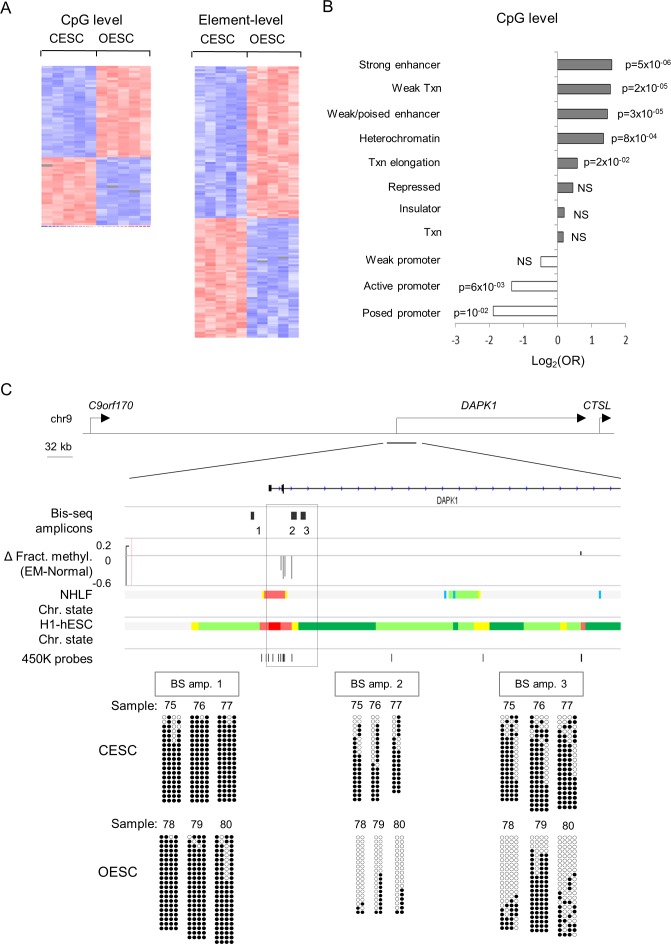
Gene-specific alterations in DNA methylation in primary ovarian endometriosis stromal cells (OESC) compared to control endometrial stromal cells (CESC), chromatin state enrichment analysis of DM genes in endometriosis, and bi-seq validation for *DAPK1*. **A)** Heat maps of gene-specific methylation changes in endometriotic stroma cells vs. controls. Supervised hierarchical clustering of the 450K methylation BeadChip data analyzed at the CpG level (FDR q-values < .05 and absolute difference in fractional methylation (Δmethyl.)>.15) and at the element-level (q-values < .05 and Δmethyl.>.15 for multiple CpG segment and>0.1 for single CpG segment) are shown. Biological samples are on the x-axis and differentially methylated loci are on the y-axis, with relative hypermethylation and hypomethylation indicated by the color scale. The fractional methylation values for each CpG are centered and standardized to have mean 0 and standard deviation 1. The red color represents a methylation level above the mean methylation of the CpG across all samples, the white color represents mean methylation and the blue color represents methylation lower than the mean. **B)** DM CpGs are enriched in enhancers but depleted in promoter regions. For each chromatin state, enrichment and under-representation are symmetrically visualized using log_2_(OR). **C)** Validation and mapping of the DMR in *DAPK1* using bis-seq. A map of the *DAPK1* gene showing hypomethylation in the promoter region is given on the top. The DMR overlaps an active promoter region (color coded in red) flanked by a strong enhancer (yellow). Bis-seq amplicons for validation and mapping of the DMR are indicated by the numbered rectangles. The bis-seq data (bottom panel) is visualized by the circles representing consecutive CpGs with black circles indicating methylated CpGs and white circles unmethylated CpGs, with each line being a unique DNA clone.

To identify changes in patterns of CpG methylation we isolated genomic DNA from early passage explant cultures of stromal cells from tissue biopsies of 5 patients with endometriosis and from parallel cultures of control uterine endometrial stromal cells from 5 individuals (OESC and CESC, respectively; **S1 Table**). We analyzed these samples on Illumina Infinium HumanMethylation450 BeadChips. With the resulting data, we first carried out a CpG-level analysis, using stringent criteria of false discovery rate (FDR) < .05 and average difference in fractional methylation (DFM)>.15 to identify a set of 68 CpGs, located in 43 genes, with strong and highly consistent DM between the cases and controls (**Fig 1A** and **S2 Table**). Supervised hierarchical clustering to produce methylation heat maps highlighted that both gains and losses of methylation occur in OESC vs. CESC (**Fig 1A** and **S2 Table**).

Since stringent correction for multiple testing in genome-wide data can lead to the discarding of some true-positive differences, and since the methylation status of multiple contiguous CpGs is thought to be important in creating or maintaining chromatin states, we used two approaches based on biological criteria to improve the identification of DM genes. First, genes with multiple DM CpGs are more likely to be true positives. Our DM criteria based on a lenient nominal p-value <0.05 require at least 2 CpGs or segments within a given gene. Second, the methylation status of multiple contiguous CpGs is more likely to be correlated and to reflect the same regulatory sequence. Focusing on CpGs in promoter, enhancer and insulator sequences, we grouped contiguous CpGs into 500 bp DNA segments to identify DM regulatory segments (among a total of 197,949 genomic segments, see Methods) For each segment, fractional methylation values of contiguous CpGs were averaged and to identify genomic segments with DM between cases and controls, we required the CpGs in a segment to have an average DFM>.10 (or DFM>.15 for segments mapping only 1 CpG queried by the BeadChips), and we again used FDR < .05, but with the correction for multiple comparisons calculated based on the number of evaluable segments. This element-level analysis resulted in a somewhat larger set of 183 CpGs (corresponding to 141 segments), in 91 genes, with DM between OESC and CESC (**Fig 1A** and **S3 Table**).

Next, we asked whether certain classes of DNA sequences and chromatin states might be preferentially affected by DM in the OESC vs. CESC comparison. Using chromatin states as defined by public data from ENCODE and related projects [25], we applied bioinformatic enrichment analyses analogous to those in our previous work [15] and found, using the DM sets from our CpG-level analysis, that the DM CpGs are over-represented in active and poised enhancer regions (Odds Ratio (OR) = 3.0; p = 5x10^-06^ and OR = 2.8; p = 3x10^-05^) but under-represented in active and poised promoters (OR = 0.4; p = 6x10^-03^ and OR = 0.27; p = 10^−02^, **Fig 1B**). This finding of enrichment of DM in enhancer elements is relevant to our analysis of the relationship of DM to DE, described in later section.

### The DM affects genes with known or suspected roles in endometriosis lesion formation

To gain insight into the functions of the genes being epigenetically regulated, we carried out manual annotations based on literature and gene ontology databases (**Table 1** and **S4 Table**), and performed gene set enrichment analysis (GSEA) and gene ontology enrichment analysis (GOEA) by overlapping the lists of DM genes identified by our stringent CpG-level and regulatory element-level criteria with gene sets using Broad Institute-GSEA website (www.broadinstitute.org/gsea) [28], [29] and with gene ontology annotation using DAVID (https://david.ncifcrf.gov/) [35, 36]. By these unbiased approaches (Methods), the GOEA was more informative than GSEA, revealing sets of DM regulatory regions, which are localized in multiple genes encoding TFs and signaling components, and showing significant enrichments in genes that control cell proliferation, nervous system development, and immunity (**S4 Table)**. GESA analysis, however, identified sets of DM regulatory regions localized in multiple estrogen-regulated genes (**S4 Table).**

**Table 1:** Examples of genes with DM and DE in OESC versus CESC.

Gene	DM CpG^a^	Rho^b^	p-value^c^	Function	Reference
***NR5A1***	6	0.972	1.15E^-05^	NR, development, steroidogenesis, reproduction	[37], [38]
***CYP1B1***	7	0.895	0.001	ENZ, steroid metabolism, proangiogenic factor	[39], [40]
***GATA4***	7	-0.979	4.34E^-06^	TF, development, sex determination	[41], [42]
***RGS5***	4	-0.921	4.32E^-04^	GTP-ase activator, vascular remodeling	[43]
***S100A4***	6	-0.950	8.74E^-05^	Oncogene, cell differentiation, motility,cell cycle	[44], [45]
***HOXA10***	10	-0.940	1.67E^-04^	TF, organogenesis, decidualization	[46], [47]
***HOXA11***	3	-0.931	0.0003	TF, decidualization, endometrial receptivity	[48], [46]
***COL7A1***	3	-0.848	0.0038	Extracellular matrix protein, PRO-regulated	[49], [50]
***OSR2***	13	-0.868	0.0024	TF, PRO-regulated, morphogenesis	[51], [52]
***DAPK1***	8	-0.958	0.0002	ENZ, cell survival, apoptosis and autophagy	[53], [54]
***TRERF1***	1	-0.951	7.92E^-05^	TF, co-activator of *NR5A1*, PRO-regulated	[55], [56]
***JAZF1***	10	-0.883	0.0016	TF, oncogene in EM stroma cancer	[57]
***WNT5A***	3	-0.946	0.0001	WNT ligand, organ development, PRO-regulated	[58], [59]
***BDNF***	1	0.900	0.005	Secreted NGF, NS development, loss of function polymorphism in endometriosis	[60], [61]
***TGFBR1***	1	0.890	0.0018	Receptor, reproductive tract integrity	[62], [63]
***ROR1***	3	0.893	0.0012	ENZ, WNT receptor, tissue morphogenesis	[64], [65]
***HAND2***	4	-0.957	5.32E^-05^	PRO-regulated, decidualization	[66], [67]
***NRP2***	4	-910	0.0016	Transmembrane co-repressor, lymphatic vessel formation	[68]
***SLC22A23***	5	-0.964	2.69E^-05^	Ion transporter, gene polymorphisms associated with EM	[69]
***SGK1***	7	0.929	0.0003	ENZ, decidualization, loss of gene function is associated with pregnancy loss	[70]

Lenient CpG DM genes showing correlation between expression and methylation were ranked based on rho correlation coefficient and methylation changes. The top 20 genes are shown. For each gene, the number of DM CpGs (^a^), the overall difference in fractional gene methylation, the number of CpGs (^b^) with correlation between DM and DE with corresponding Rho-coefficient and nominal p-value of the CpG (^c^) with the highest rank are indicated. The complete lists of DM genes showing correlation between expression and methylation are in S5A and S5B Table. The complete lists of DM genes showing correlation between expression and methylation are in S5A and S5B Table. NR-nuclear receptor, TF-transcription factor

Our manual curation of the gene lists using literature searches confirmed these conclusions (**Table 1**). These results suggest that the pathogenesis of endometriosis may involve epigenetic modulation of gene regulatory programs for neurogenesis, cell proliferation and immune responses, which are known or suspected to support ectopic lesion implantation and growth.

### Validations and extension of the BeadChip findings by bis-seq

**Fig 2:**
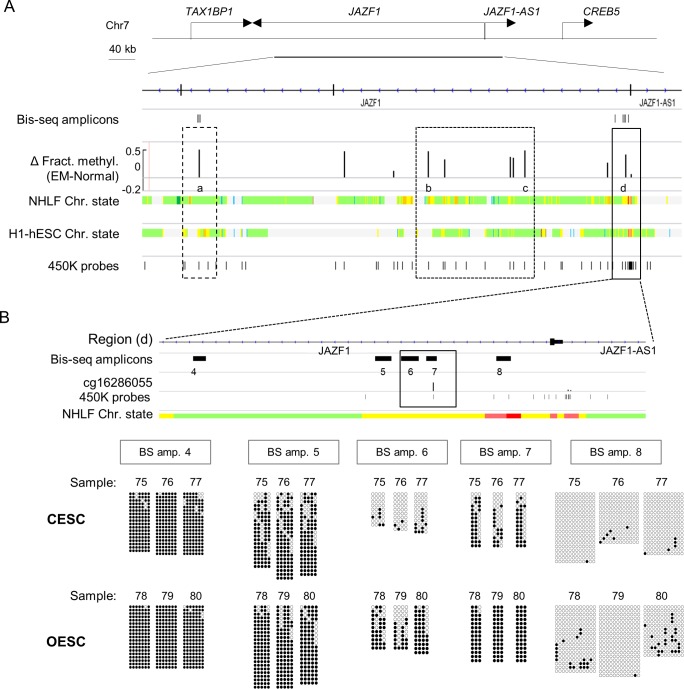
Gain of DNA methylation in the promoter and gene body of JAZF1. **A)** Map showing multiple clusters of hyper-methylated CpGs in the promoter and gene body of *JAZF1*. The dashed and plain rectangles indicate clusters of CpGs with DM overlapping multiple individual enhancer regions. **B)** Validation and mapping of the DMR (d) in *JAZF1* gene using bis-seq. The results from the Bis-seq for the amplicons indicated with number on the higher resolution gene map (top) are shown as QUMA blots (bottom). Every circle represents a single CpG. Black circles indicate methylated CpG and the white free of methylation CpG. The number of the amplicon and each individual sample ID are indicated on the top of every QUMA. The bis-seq data identify this DMR as a large 2.75 KB region spanning amplicons 5, 6 and 7.

The Beadchip assays query many CpGs, but these still represent only a small percentage in each CG-rich gene regulatory region. For example the DMR in *DAPK1* was only partially covered by the array (**Fig 1**). Similarly, for *JAZF1*, the DMR and flanking regions are queried at only by 1 CpG in the BeadChip assay (**Fig 2B** and **S1 Fig**). Further, since SNPs in the probe binding sites and a few ambiguously mapping probe sequences can complicate the BeadChip data interpretation, it is desirable to perform validations using bis-seq, which can reveal the pattern of methylation across multiple contiguous CpGs including and surrounding the DM CpGs queried by the Beadchips. Determining the boundaries of the DMRs in this way is crucial for understanding the biological mechanisms and consequences of DM. Using bis-seq, we confirmed hypomethylation in the promoter of the *DAPK1* gene in OESC and extended the results to additional DM CpGs. This DMR was localized to the downstream part of the *DAPK1* promoter **(Fig 1C)**. At least four DMRs in the *JAZF1* gene were identified in our analysis, located in strong enhancer sequences and near the promoter of the gene (**Fig 2A**). We validated and extended the findings of DM in two of these regions using bis-seq. The first of these DMRs, marked as region “d” in **Fig 2B**, is located in *JAZF1* intron 1 within an ENCODE-defined enhancer element. Our bis-seq data identified it as a large DMR with gain of methylation in endometriotic stromal cells vs. control endometrial stromal cells. Bis-seq of a second DMR in the *JAZF1* gene, located in a gene body enhancer element, was also confirmed, extending the BeadChip results by coverage of additional DM CpGs (**S1 Fig**).

Since endometriosis is associated with abdominal pain and nerve fiber outgrowth within the lesions, we further examined the CpG methylation changes in the *BDNF* gene, which codes for a secreted nerve growth factor that was shown to be elevated in tissue samples of patients with endometriosis vs. controls [71] and for which a loss of function polymorphism has been associated with endometriosis-related infertility [72]. Our bis-seq data for this gene validated and extended the BeadChip findings of gains of methylation in the OESC samples (**S2 Fig**). Similarly, we validated and extended our 450K findings of gain of methylation in a CpG overlapping the last exon of the *TGFBR1* gene, encoding an important signaling receptor, with bis-seq extending the 450K data by revealing gains of DNA methylation at multiple CpGs in OESC (**S3 Fig**).

### Analysis of 5mC and 5hmC in endometriosis lesions and stroma cells

**Fig 3:**
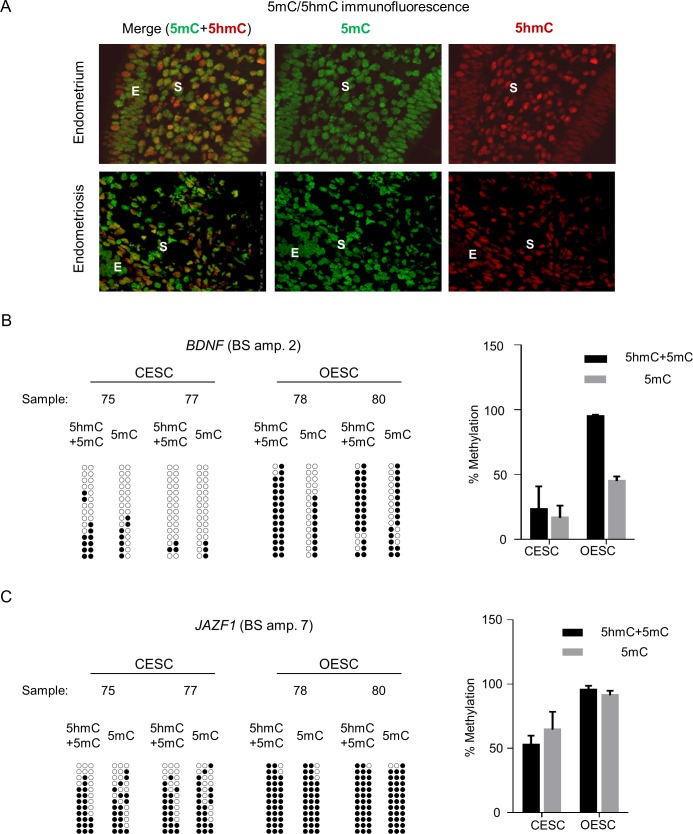
Analysis of 5mC and 5hmC in endometriosis lesions and stroma cells. **A)** Immunofluorescence analysis of the levels of expression of 5mC (green) and 5hmC (red) in tissue samples of women with endometriosis and controls, showing disease-dependent loss of 5hmC in the epithelial but not in the stromal cell compartment. Representative photos from a total number of n = 2 control and n = 2 endometriotic tissue are shown. **B)** The results of the standard and oxidative bis-seq for *BDNF* in endometriotic and control stromal cells represented as QUMA plots (left) and as bar graph (right). The QUMA plot and bar-graph show the percent methylation assessed by standard bis-seq (which scores indistinctly 5hmC and 5mC) and by modified bis-seq (which scores 5mC only) in control stroma and endometriotic stroma cells. The contribution of 5hmC in endometriotic and control stroma is inferred from the difference between the percent methylation at each CpG in the standard bis-seq reactions (5hmC + 5mC) and the percent methylation in the modified bis-seq reactions (5mC). **C)** The data of *JAZF1* standard and oxidative bis-seq in endometriotic and control stromal cells represented as QUMA plots (left) and bar-graph (right) are shown.

To examine possible contributions of changes in the “sixth base”, 5hmC, we first used IF to examine the relative amounts of 5mC versus 5hmC in endometriotic lesions. Strikingly, at the whole tissue level we found an obvious loss of 5hmC in the epithelial cell compartment of endometriosis, compared to epithelial cells in control endometrium, with no loss of global 5hmC in the endometriotic stroma cells (**Fig 3A**). Next, we tested two DM loci for the detailed pattern of 5hmC using both standard and oxidative bis-seq applied to OESC and CESC samples. As shown in **Fig 3B and 3C**, both types of modifications contributed to the net DM, with the relative contributions differing between the two genes: a strong relative contribution of 5hmC was seen in the *BDNF* gene (**Fig 3B)** with concurrent and equal gains of both 5mC and 5hmC, while the enhancer DMR in the *JAZF1* gene **(Fig 3C**) show little or no contribution. These results show that while the precise balance between these two DNA modifications is different between different genomic loci, when 5hmC is detected, the direction of the change in 5hmC can parallel that of 5mC.

### Effects on gene expression are more frequent when the DM is localized to enhancer sequences

Differential mRNA expression (DE) is obviously an important functional readout of DM, but in most epigenetic studies only a subset of genes with DM are found to show DE. Thus it is thought that many genes with DM are “bystanders”, and the search for genomic and epigenomic features that distinguish these bystander loci from the biologically relevant loci whose expression is affected by DM is a fundamental question. To investigate this situation in endometriosis we performed RNA-Seq on 5 control and 4 endometriotic stromal cell samples for which RNA was available from the same early passage cultures as had been profiled for DM (**S1 Table**). The results showed DE of a large group of genes in OESC compared to CESC, with GSEA revealing alterations in gene networks with roles in apoptosis, cell differentiation, neuronal development, response to estrogens, and genes coordinately up-regulated in a compendium of adult tissue stem cells **(S4 Table)**, and GOEA revealing significant enrichment in cell survival, immune responses, cell migration, neuronal differentiation and hypoxia pathways **(S4 Table).**

Using the RNA-Seq data, we identified CpGs and genomic segments with strong overall correlations between methylation and expression (p<0.005 and correlation coefficient>0.7), independent of case-control status and restricting the analysis to genes expressed above a standard cut-off (average FPKM>1 in at least one of the groups). Using these criteria, we identified 1623 and 1105 genes with positive or negative overall correlations between methylation and expression at the CpG-level and element-level, respectively. Among the genes with negative correlations (hypermethylation correlating with reduced expression), 54% showed it at the CpG-level and 58% at the element-level.

Next, we brought forward the lists of DM genes identified by lenient CpG-and element-level case-control comparisons for overlap with this methylation-expression correlation list. We found 306 (CpG-level) and 239 (element-level) genes with DM for which methylation correlates with expression (**S4A Fig**, **S5 Table** and **Methods**). Thus, 19% of genes with DM showed correlations between methylation and expression. While this fraction is a minority of the DM genes, it nonetheless represents a significant enrichment over random expectation (O.R. 5.8, p = 5x10^-185^). Among these DM and DE genes, the *JAZF1* and *DAPK1* DM regions showed a strong correlation between the fractional methylation and the gene expression (**S4B and S4C Fig**). Q-PCR validation analysis for the *JAZF1* gene showed a significant 2.3-fold (p = 0.003) down-regulation of the levels of *JAZF1* expression in OESC versus CESC. Based on genomic annotations the *JAZF1* locus contains an antisense transcript that arises from a promoter region located in intron 1 of the protein coding gene, and our results from Q-PCR of DNAse-treated RNA samples using primers specific for the main antisense transcript suggest that the DM in OESC does not markedly affect the expression of the apparently spliced and non-translated *JAZF1-as* RNA **(S5A Fig).**

Although a large number of other CpGs that showed overall correlations between methylation and expression in our combined OESC plus CESC sample sets did not pass the stringent cutoffs for DM in our case-control comparisons, they showed on average an absolute difference of fractional methylation of 0.096, i.e. DM that is sub-threshold by our criteria. Notably, *TGFBR1* and *BDNF* showed a strong correlation between expression and methylation (p = 0.0018, rho correlation coefficient = 0.89 and p = 0.005, rho = 0.9, respectively) but did not pass our DM criteria at FDR < .05 since only 1 of the 450K-queried CpGs in each gene showed strong DM (DFM = 0.4, nominal p-value = 0.002 and DFM = 0.4, nominal p-value = 0.007, respectively (**S4D and S4E Fig**). Nonetheless, as noted above using bis-seq we confirmed, for both of these gene regions, DM not only in the 450K-queried CpG but also in several contiguous CpGs (**S2 Fig** and **S3 Fig**). Consistent with previously reported positive relationship between intra-genic hypermethylation and increased gene expression, the hypermethylation of the DM region in intron 2 of the *BDNF* gene was associated with up-regulation of *BDNF* mRNA expression in OESC (6-fold, p = 0.008 by Q-PCR) (**S4B Fig**) and with increased protein secretion (8.5- fold by ELISA; p = 0.006, **S5C Fig**) by these endometriotic stromal cells.

We next used a series of bioinformatic analyses to seek mechanistic explanations for the observed overlap of some but not all DM regions with DE in the EOSC versus CESC comparison. We first tested for the effect of increasing stringency for DM (number of DM CpGs in the gene or regulatory element and p-value in the case-control comparison) and found stronger enrichment for correlation of DM with DE with increased strength of the DM (**S6 Fig**). When examined at the regulatory element level, we found that CpGs with correlations between methylation and expression were enriched in enhancers but depleted in promoters (OR = 2.6, p = 7x10^-142^ and OR = 0.6, p = 2.3x10^-42^, respectively, **S7 Fig**). Since we used the entire set of genomic elements as the denominator for our enrichment analyses, this strong finding is only partly explained by the overall enrichment in enhancers that we had observed among DM CpGs. The simplest mechanistic interpretation is that it reflects a greater methylation-sensitivity of the function of enhancer regions, compared to promoter regions, particularly when the changes in fractional methylation occur in a modest range of 0.1–0.4, as is true in this dataset and in most other methylation datasets from epigenomic studies of non-neoplastic diseases.

To better understand this observation, we compared the methylation distribution between CpGs correlating and those not correlating with expression. The methylation distributions for CpGs not correlating with expression were, as expected, bimodal in enhancers and insulators but unimodal (low methylation) in promoters, which often correspond to CG-islands that are generally protected from methylation. The methylation distributions of CpGs correlating with expression showed an enrichment of the intermediate methylation levels in OESC for all the tested regulatory elements. We also compared the methylation levels between DM CpGs correlating with expression and DM CpGs not correlating with expression. Overall, DM CpGs correlating with expression were associated not only with greater methylation changes, but also with a more complete unmethylated or methylated status in the control (CESC) cell populations (**S6 Table**). Since intermediate net methylation levels can often reflect heterogeneity of methylation within cell populations, a reasonable hypothesis is that DE is more likely to be detectable when DM occurs uniformly across the cell population.

In summary, we observed (i) a statistically significant enrichment among DM loci of CpGs where methylation correlates with expression, supporting a functional role of DM in endometriosis and, (ii) enrichment of DM CpGs correlating with DE in enhancer elements and depletion in promoters, suggesting that functionally relevant DM in endometriosis stromal cells occurs more frequently in dynamic than in primarily constitutive regulatory elements.

### The *HOXA* gene cluster, *TBX3*, *NR5A1*, *DAPK1*, *RGS5* and members of the *GATA* family of transcription factors show DM and DE both in our data and in a prior study

Previously, Dyson et al. [21] performed methylation analyses on OESC compared to CESC samples, obtained at a different medical center independently of our cases and controls, using 450K BeadChips. With a very lenient cutoff requiring DFM >0.15 with no p-value criterion, they found 45,425 DM CpGs located in 9,021 genes. Given their very large DM set, as expected, 96% of our DM CpGs are included in that set. In the same report, those investigators went on to characterize gene expression using microarrays and then performed ANOVA interaction modeling, yielding a much smaller list of 403 genes that showed both DM and DE. Our list of DM genes with significant correlations between DM and DE contains 77 of those genes. Important examples of genes with concurrent findings in both studies are the *HOXA* gene cluster, members of the GATA family of TFs (*GATA2*, *GATA4* and *GATA6*), and *TBX3*, which encode transcription factors (TFs) that specify cellular identities in development, *NR5A1*, coding for a TF that plays a role in endometriosis by deregulating steroid signaling [73, 74], and *DAPK1*, encoding a protein kinase that regulates cell survival and apoptosis [75]. Epigenetic changes in these genes, with downregulation and hypermethylation of *GATA2* and hypomethylation and activation of *GATA6*, have been postulated to be involved in progesterone resistance and altered estrogen responses in endometriosis [21]. The *RGS5* gene codes for a cytoskeleton regulating protein that can mediate an epithelial-mesenchymal transition in cancer cells and was previously found to be differentially expressed and up-regulated in endometrial mesenchymal stem cells [5, 76].

### Examples of novel differentially methylated and expressed genes in endometriosis stromal cells

**Fig 4:**
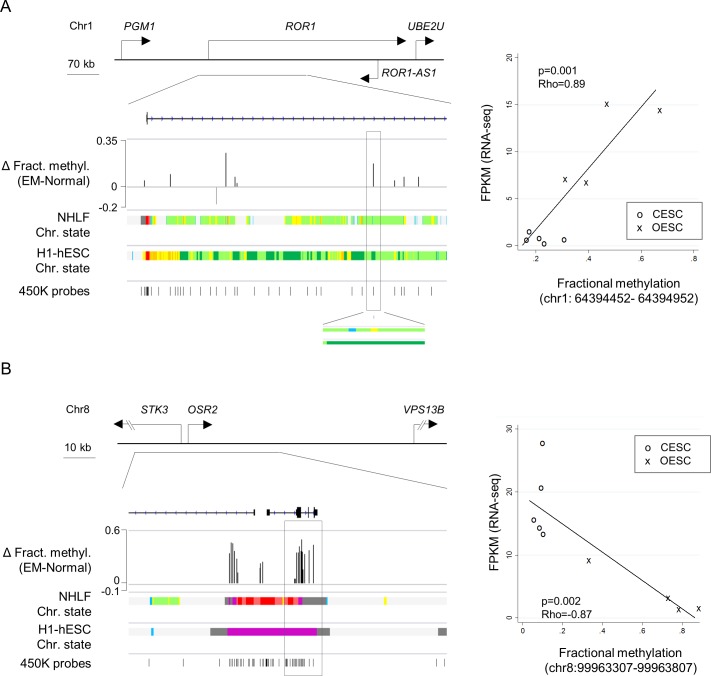
DNA methylation changes at regulatory DNA elements in *ROR1* and *OSR2* correlate with gene expression. **A)** Map and XY graph of the DMRs in *ROR1*. Multiple hyper-methylated CpGs are identified in the gene body. Differences in fractional methylation between OESC and ESC are indicated for all CpGs with nominal p-value<0.05 in the bar graph. Chromatin state in NHLF and H1-ESC cell lines (ENCODE project) are color coded as described in the USCS browser. A positive correlation between methylation and expression is observed at cg19267457 (rectangle), overlapping an enhancer region (color coded in yellow). The XY graph shows expression levels, assessed by RNA-Seq and estimated by FPKM values, as a function of the fractional methylation assessed by Illumina BeadChip arrays at cg19267457. **B)** Map and XY graph of the DMRs in *OSR2*. Strong hyper-methylation is observed in and upstream *OSR2*. The DMRs overlapped a dynamic promoter, active in differentiated cells and poised in embryonic stem cells (color coded in red or purple, respectively). The cluster of DM CpGs in the gene (rectangle) shows a strong negative correlation between methylation and expression. The XY graph shows *OSR2* expression level against the fractional methylation of the CpG with the highest absolute rho correlation coefficient.

Examples of interesting novel epigenetically affected loci that showed DM and DE in our series include genes coding for TFs (*OSR2*, *JAZF1*), extracellular matrix proteins (*COL7A1*), transporter proteins (*SLC22A23*), receptor proteins (*TGFBR1*, *ROR1*) and secreted signaling proteins (*WNT5A*, *BDNF*). The transmembrane tyrosine kinase receptor ROR1 has been shown to inhibit apoptosis, potentiate EGFR signaling and to induce epithelial to mesenchymal transitions [36, 77]. A mouse genetic model with disruption of Wnt5a-Ror1 non-canonical Wnt signaling identified this gene as an important factor in embryo implantation, decidualization and placentation. In our data *ROR1* shows hypermethylation in OESC, with the DMR overlapping an enhancer element (**Fig 4A**, **Table 1**, **S3B Table** and **S5B Table**), which positively correlates with gene expression (Rho = 0.89, p = 0.001) in OESC. The gene locus also hosts a non-coding RNA antisense transcript with unknown function. The *OSR2* TF gene has been shown to be progesterone-regulated in endometrial stroma, where it may regulate decidualization. We found promoter hypermethylation and transcriptional down-regulation of this gene in OESC vs. CESC, with a high negative correlation between DM and DE (Rho = 0.87, p = 0.002, **Fig 4B**).

Another very interesting and potentially functionally important DM and DE gene identified in our data is *JAZF1*, which encodes a TF [78] and also hosts a small nuclear RNA U6 involved in spliceosome assembly and a long non-coding antisense RNA transcript. The gene is a “hotspot” for chromosomal translocations resulting in gene fusions in endometrial stromal tumors [79] and chimeric *JAZF1-JJAZ1* mRNA transcripts, resembling the gene fusion in cancer, have been found to be produced by physiological trans-splicing in human cells. *JAZF1* is also expressed in normal endometrial stroma, with higher abundance in early proliferative and late secretory phases of the menstrual cycle [80]. In our data this gene is broadly hypermethylated at multiple intragenic and flanking positions and its expression is down-regulated in OESC compared to control CESC (**Table 1** and **S3B Table** and **S5B Table**). Lastly, in our data the *WNT5A* gene showed concurrent gain of promoter methylation and transcriptional down-regulation in the OESC samples (**Table 1**). WNTs are secreted signaling molecules that control a variety of biological processes such as cell polarity, cell differentiation, proliferation, and survival [81]. In human endometrium, *WNT5A*, encoding a non-canonical WNT signaling protein, acts as pro-survival protein during decidualization [82], so this gene is a compelling candidate for a functional role in endometriosis.

### Functional testing: effects of the hypomethylating drug 5aza-dC

**Fig 5:**
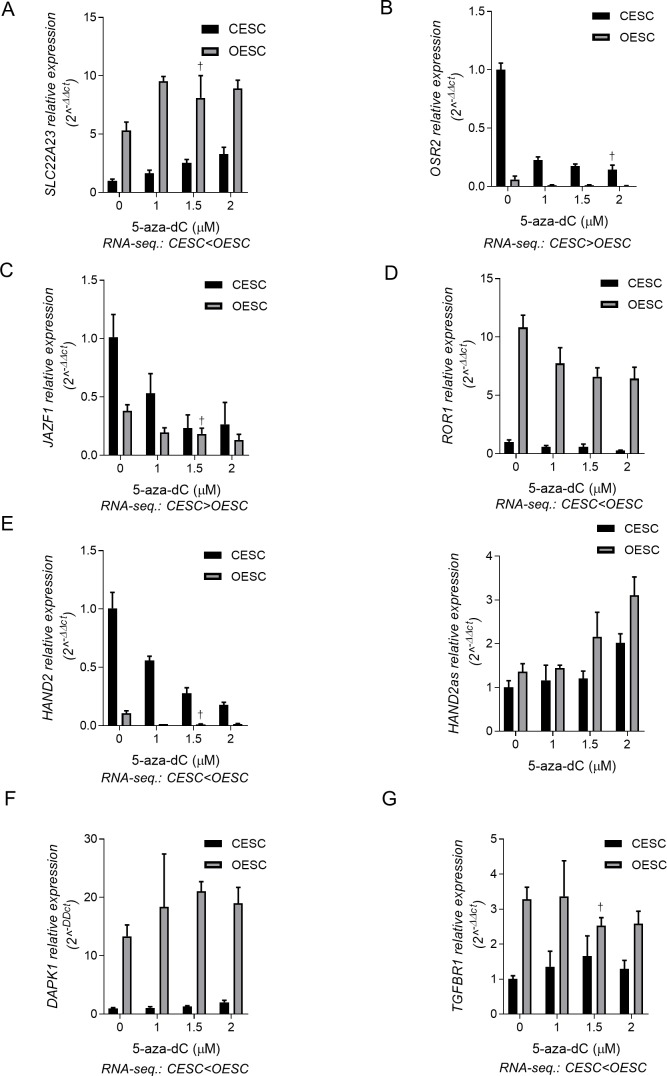
Validation of RNA-sequencing by Q-PCR and effects of 5Aza-dC on a subset of DM and DE genes in CESC and OESC. This figure shows average gene expression levels in CESC and OESC, from two or three biological replicates, after treatment with 5aza-dC for 72 hrs. All values were normalized to the mean of the non-treated CESC controls (0) set to 1. **A)** The relative expression of *SLC22A23* under treatment with 5aza-dC increases in CESC as well as in OESC. In CESC, the increase in expression is dose dependent. **B)** The relative expression levels of *OSR2* are decreased in CESC as well as in OESC under all tested doses of the demethylating agent. **C)** Treatment with 5aza-dC is associated with decreased relative expression levels of *JAZF1* in both control and endometriotic stromal cells. **D)** Although in both CESC and OESC the *ROR1* expression levels decrease under 5aza-dC treatment, in CESC the effect on gene expression is dose dependent and more pronounced compared to the endometriotic cells. **E)** While the *HAND2* gene relative expression levels decrease after demethylation treatment as shown in the left bar graph, the *HAND2as* levels change in the opposite direction; the expression levels are increased in both CESC and OESC, especially at the highest level of 5aza-dC. *DAPK1* **(F)** and *TGFBR1* **(G)** expression levels are not influenced by the drug in CESC or in both CESC and OESCs, respectively. For direct comparison of Q-PCR and RNA-seq data, the differences in expression between CESC and OESC, identified by RNA-seq method are indicated below each graph. CESC, control endometrial stroma cells, OESC, ovarian ectopic endometriosis stroma cells. In each graph, † indicates conditions with biological duplicates.

To examine the effects of altering DNA methylation on the functional readout of gene expression, we treated the stromal cells with low (sub-cytotoxic) concentrations of the DNA hypomethylating agent 5Aza-dC for several days (see Methods) to permit replication-dependent genomic hypomethylation, followed by Q-PCR analysis of mRNA expression of 7 of the DM/DE genes (**Table 1**). We observed significant effects of the hypomethylating drug on the expression of most of these genes, but with the directions of the effects differing among the tested genes. *SLSC22A23* codes for a membrane protein that transports organic ions and haplotypes at this locus have been associated with endometriosis-related infertility [69]. In our data the DM in this gene is localized both in promoter and enhancer sequences. In CESC and OESC treated with 5aza-dC, the relative levels of *SLC22A23* expression were 3.3-fold (p = 0.015) and 1.68-fold (p = 0.0035) higher compared to non-treated cells (**Fig 5A** and **Table 2).** The DMRs in the *OSR2* gene were likewise located in both promoter and enhancer elements (**Fig 5B, S3 Table** and **S5 Table**), but in contrast to the *SLC22A23* gene treatment with 5Aza-dC led to down-regulation of *OSR2* mRNA expression in both cell types (**Fig 5, S8A Fig** and **Table 2**). The *JAZF1*, *HAND2* and *ROR1* genes are all complex loci hosting non-coding RNA transcripts. DM at *JAZF1* and *ROR1* is widespread and affects several intragenic enhancers and insulator sequences, while DM in *HAND2* was localized to the promoter region (**S3B Table** and **S5B Table**). Gains of DNA methylation in *JAZF1* and *HAND2* in OESC compared to CESC are associated with transcriptional down-modulation, but somewhat unexpectedly under treatment with 5Aza-dC these two genes showed downregulation of their expression in both cell types **(Fig 5, S8B Fig** and **Table 2)**. Down-modulation of expression of the *HAND2* protein-coding gene by the hypomethylating treatment was associated with an increase in *HAND2as* expression (2- fold increase, p = 0.004 for CESC and 2.3-fold, respectively, p = 0.008 for OESC) (**Fig 5E**, **S8B Fig** and **Table 2**), suggesting a possible mechanism for the observed “paradoxical effects”. *ROR1* on the other hand is hypermethylated and up-regulated in OESC vs. CESC. This suggests that the increased methylation in OESC may be acting via its effect on intragenic insulator sequences. The hypomethylating treatment decreased the mRNA levels of this gene in both types of cells, though more profoundly in CESC (**Fig 5D**). Here it is important to note that the baseline levels of *ROR1* expression in CESC were very low, showing mean FPKM = 0.8 by RNA-Seq, and Q-PCR CT-values of 32 cycles and higher (**Table 2**). This result is in agreement with previous observations where it was found that in human adult tissues the protein is either absent or expressed at low levels. High levels of ROR1 were found in solid and blood malignancies [83][84], which makes it a possible diagnostic and targeted therapy marker. Overall, we could confirm the RNA-seq data using Q-PCR for all seven genes (**Table 2**). We further showed that the decrease in methylation at the promoter of *DAPK1* in OESC compared to CESC was associated with upregulation of gene expression. Decrease of DNA methylation at the promoter of the *DAPK1* gene in CESC under 5aza-dC treatment resulted in activation of mRNA expression (2- fold increase, p = 0.02) (**Fig 5F** and **Table 2**). This result, together with our observation of decreased methylation levels around the promoter of the gene in OESC compared to CESC, suggests that the increased levels of expression of the gene in non-treated OESC (Fig 5F and Table 2) are due to loss of methylation in this region. Further, we asked whether changes in the levels of DNA methylation at the promoter in OESC can affect gene expression. As shown in **Fig 5F**, 5aza-dC treatment resulted in activation of mRNA expression in OESC, which was lower than the effect of the drug observed in CESC (1.4-fold, p = 0.05). Overall, these data show that the levels of DM within the promoter region of *DAPK1* gene account for the degree of transcriptional activation rather than regulating the “on-off” expression state. Lastly, the levels of expression of *WNT5A* (**Table 2**) and *TFBR1* (**Fig 5G**) were not affected by the global changes in DNA methylation in our experimental conditions, suggesting more complex mechanisms of transcriptional regulation of these genes.

**Table 2:** Validation of RNA-sequencing by Q-PCR for seven DM and DE genes and effects of the hypomethylating drug 5aza-dC on their expression.

	Control endometrial stroma cells					Ovarian endometriosis stroma cells				
Gene	AVG.RPKM	AVGCt	AVG. ΔCt^a^	AVG. DM^b^	Fold change^c^	AVG. RPKM	AVG. Ct	AVG. ΔCt^a^	AVG. DM^b^	Fold change^c^
***JAZF1***	40.6	23.7	3.5	0.249	3.807^d^	17.8	24.0	4.9	0.628	2.889^d^
***ROR1***	0.9	31.4	7.6	0.353	3.556^d^	10.9	28.3	4.1	0.437	1.682^d^
***OSR2***	18.5	20.3	6.4	0.133	9.047^d^	3.9	24.6	11.2	0.503	4.795^d^
***SLC22A23***	0.4	25.2	5.1	0.431	3.271^d^	8.0	28.4	2.5	0.538	1.681^d^
***HAND2***	18.3	23.3	1.8	0.076	5.657^d^	4.5	26.3	5.0	0.370	7.086^d^
***HAND2as***	ND	27.0	3.9	0.076	2.002^d^	ND	27.5	3.5	0.379	2.286^d^
***DAPK1***	0.2	28.9	11.1	0.637	2.015^d^	17.5	22.2	7.4	0.478	1.426^d^
***WNT5A***	233.9	19.9	-4.5	0.280	NC	66.8	22.1	-1.4	0.532	NC

The levels of expression of DE genes in control and endometriotic stromal cells identified by RNA-seq and validated by Q-PCR, and overall levels of DM, are shown. For each gene, the RPKM values from RNA-seq., the row averages Ct-values for non-treated (0) controls for CESC and OESC, corresponding normalized ΔCt values, the averaged overall DM at the CpG level for each gene, and the difference in the expression between non-treated (0) controls and cells treated with 2μM 5aza-dC, are listed. RPKM- reads per kilobase per million

^a^—averaged normalized to housekeeping gene ΔCt values

^b^- averaged overall gene DM at CpG-level

^c^- expression given as Fold change under 2μM 5aza-dC treatment vs. non-treated control

^d^- statistically significant: p-value <0.05; ND-not determined; NC-no change

While the directions of the effects of the hypomethylating agent differed among the genes tested, the effects on gene expression correlated with the levels of baseline methylation. Except for the *DAPK1* gene, in CESC and OESC cells genes with widespread DM show more profound changes of their expression when the overall level of gene methylation was low, compared to genes with overall high baseline levels of methylation (**Table 2**).

### *WNT5A* promoter hypermethylation correlates with reduced expression of WNT5A protein in stromal cells at the whole tissue level

**Fig 6:**
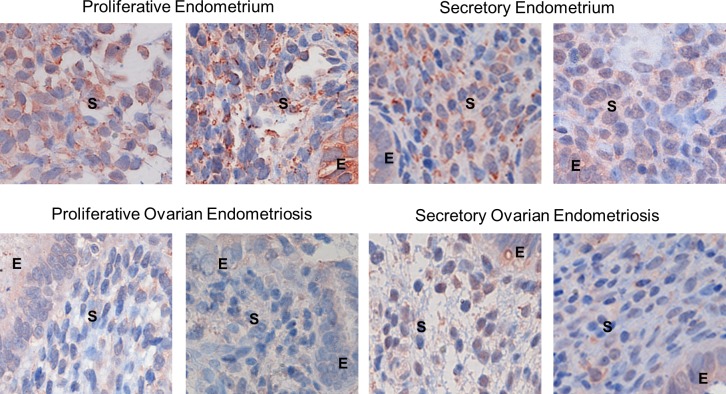
WNT5A protein expression at the whole tissue level. Immunostaining for the WNT5A protein was done on FFPE sections of endometriotic (n = 14) and normal endometrial tissue (n = 14) samples. Representative pictures from control endometrium and endometriotic lesions at proliferative and secretory phase of the menstrual cycle are shown. The stromal = S and epithelial compartments = E are indicated. The results indicate down-regulation of the levels of WNT5A in the stroma of endometriosis vs. control endometrium, independent of the cycle phase.

Arguing for relevance of our findings from the stromal explant cultures to the in vivo situation, a number of the genes that we found to be differentially expressed in OESC compared to CESC stromal cells, including *GATA6*, *HOXA11* and *TBX3*, among others, were also found to be differentially expressed in Gene Chip expression array data from whole endometriotic lesions in the study of Crispi et al [85]. To further investigate this question, we screened antibody reagents for several of the top-ranked DM/DE genes. We found a useful antibody against WNT5A and used it to perform IHC on tissue sections of ovarian ectopic endometriotic lesions and control uterine endometrium. As shown in **Fig 6** endometriosis stromal cells have significantly lower intensity of WNT5A signal and therefore, lower levels of stromal WNT5A compared to normal endometrium stroma. When looking at the levels of the normal stromal WNT5A expression in the context of the menstrual cycle phases we observed, high cytosolic expression of the protein in proliferative stroma and lower cytosolic signal in secretory phase endometrium stroma, where, a small number of endometrial stromal cells showed perinuclear WNT5A staining. No significant difference was seen in the levels of expression of WNT5A in epithelial cell compartment where the protein showed cytosolic localization.

## Discussion

Results from this comprehensive epigenomic and expression profiling study show that stromal cells of endometriosis lesions have altered patterns of DNA methylation, compared to stromal cells in normal endometrium; that these changes affect genes with roles in cell signaling, proliferation and migration, nerve development, and immunity; and that some but not all of the genes with DM also show the functional readout of altered mRNA expression (“DM+DE genes”). Using a new and broadly applicable approach for segmenting the 450K Methylation Beadchip data according to regulatory elements, we find that the DM CpGs are over-represented in active and poised enhancer regions and under-represented in active and poised promoters. Using bis-seq we validated and extended the array-based data to larger numbers of CpGs, revealing strong DMRs in regulatory elements of several of the DM genes including *JAZF1*, *DAPK1*, *BDNF* and *TGFBR1*. In conjunction with the methylation profiling, our RNA-seq data address the important general problem of incomplete correlations of DM with altered gene expression (the “DM+DE paradox”)—showing that the likelihood of DM correlating with DE increases with the extent of the DM and with its location in enhancer elements, without an enrichment of expression-correlated DM in promoter regions. These results add to an evolving picture in which the changes in DNA methylation that are associated with transcriptional changes in development and disease occur more frequently in non-promoter regulatory elements [86–88].

In our study we also assessed the relative contribution of 5mC and the “sixth base”, 5hmC. We found that global 5hmC is more abundant, relative to 5mC, in endometriosis stromal cells compared to the epithelial cell population, and is slightly decreased in endometriotic versus normal stroma. Using oxidative bis-seq we found that for the DM region in *BDNF* the changes in 5hmC parallel those in 5mC while in *JAZF1*, little or no 5hmC was detected in both OESC and CESC. Our finding of more global 5hmC in the stromal compartment compared to the epithelial compartment is reminiscent of our prior observations in a stroma-rich cancer model [24] and of results obtained by other groups who compared stromal to epithelial cells in other settings [89]. Moreover, the finding of parallel, not discordant, changes in 5mC and 5hmC is similar to observations that we and others have made in other human diseases [15], [90], supporting the idea that these two marks can sometimes act together, not antagonistically, in regulating gene expression [89].

Using GSEA and GOEA enrichment analysis we identified DM gene sets enriched in genes controlling cell proliferation, nervous system development, immunity and estrogen responsive genes. Our annotations of DM+DE genes using literature searches revealed a mechanistically interesting and potentially clinically relevant connection of some of these genes to progesterone responses. These results suggest that DM affects genes with known or suspected roles in endometriosis lesion formation.

Examples of DM+DE genes that are known to be progesterone regulated include *OSR2*, *SGK1*, *HAND2* and *WNT5A*. Consistent with known progesterone resistance of the lesions, *OSR2*, *HAND2* and *WNT5A* show gains of methylation and reduced expression in endometriotic stroma vs. controls. Previously, the essential mediator of the early decidual response *HAND2* was identified as a specific target of the GATA2 transcription factor [21], and hypermethylation of this gene is a common and crucial alteration in endometrial cancer. In addition, a knock-out of *Hand2* in uterine tissue of mice induced atypical endometrial hyperplasia as a function of age [66]. These observations suggest that epigenetic silencing of this gene in ectopic endometriotic stroma may be linked to increased cell proliferation.

Another interesting DM+DE gene is *SGK1*. Genetic studies in mice and targeted knockdown of the gene in primary human CESC revealed that *SGK1* is an important factor for decidual cell survival, while relative *SGK1* deficiency sensitized these cells to oxidative cell death [70]. Recently, *SGK1* was found to be overexpressed in endometriosis associated with regulation of ectopic stromal cell survival [91, 92]. This finding is in line with our observation that the gene is hypomethylated and overexpressed in endometriotic stroma cells.

It is known that endometriotic lesions release several pain-mediating substances such as prostaglandin E2/F2, histamine, kinins, nerve growth factor (NGF), *BDGF* and different IL, which can activate peritoneal nociceptors [93]. Estrogen is locally produced by ectopic lesions and its inhibitory effect on sympathetic nerve fibers is in part controlled by the upstream actions of ER- alpha [94], which leads to activation of expression of *NGF* and *BDNF* [95]. Thus, besides the neurotrophic properties of the endometriotic lesion itself, neuromodulation is also a hormone-dependent phenomenon. Here we have found that gain of methylation at 3 ‘sequence of the *BDNF* gene is functionally linked to up-regulation of gene transcription and protein secretion by endometriotic stromal cells, suggesting a role for epigenetic gene regulation in the mechanisms of pain generation in endometriosis.

As noted above, among the DM+DE genes passing our stringent statistical criteria are examples associated with biological processes relevant to endometriosis pathogenesis and progression such as immune responses (*TGFBR1*), neurogenesis (*BDNF*), cell proliferation (*JAZF1*) progesterone responses (*ORS2*, *HAND2*, *SGK1*), and cell signaling (*DAPK1*). The DAPK1 kinase induces cellular apoptosis in response to internal and external apoptotic stimulants and inactivation of the gene by promoter hypermethylation was associated with a broad range of human cancers. Here we identified this gene as *hypo*methylated and highly expressed in endometriotic stroma cells, suggesting that in endometriotic stroma, the DAPK1 kinase may be acting in ways other than through a pro-apoptotic function. Of note, several studies reported that DAPK1 mediates pro-inflammatory signaling downstream of TNF-alpha, LPC, and other cytokines (reviewed in [96]). As endometriosis is a chronic inflammatory disorder where the immune surveillance is impaired due to local production of pro-inflammatory cytokines including TNF-alpha, IL-1, IL-6 and IL-8 and partially originating from ectopic lesions stromal cells [35], we propose that DAPK1 in endometriotic stroma may be involved in cellular processes associated with immunity.

Prominent in our list of DM+DE genes is *JAZF1*, encoding a nuclear protein and transcriptional regulator [78] that is affected by chromosomal rearrangements in endometrial stromal tumors [79]. A chimeric *JAZF1-JJAZ1 RNA*, resembling the gene fusion in cancer, was also found to be expressed within normal endometrial stroma with higher abundance in early proliferative and late secretory phases of the menstrual cycle, where the fusion RNA transcript is a product of a rare trans-splicing event [80]. In our study this gene was hyper-methylated and its expression was down-regulated in OESC compared to CESC, suggesting an intriguing link between endometriosis and a type of endometrial neoplasm.

Supporting functional relevance of the DM in endometriotic stroma cells, treatment of stromal cells with the hypomethylating drug 5aza-dC led to activation of *DAPK1* and *SLC22A23* and repression of *HAND2*, *JAZF1*, *OSR2*, and *ROR1* mRNA expression and IHC analysis of tissue samples revealed a difference in protein expression of WNT5A in CESC and OESC. The directions of the effects of the hypomethylating agent differed among the genes tested, presumably due to different functions of the DM elements in each locus. Arguing for non-randomness of these effects we found that for genes with widespread DM—*JAZF1*, *SLC22A23* and *OSR2* –the 5aza-dC produced stronger changes in expression in cells where the starting level of gene methylation was low, compared to cells with a high starting level of methylation, which would be predicted to be more refractory to the drug effects.

How do our findings relate to prior studies of epigenetic alterations in endometriosis? Altered DNA methylation of several genes in endometriotic lesions has been previously reported from candidate gene studies, and from several genome-wide studies (**S9 Table**). In particular, DNA methylation arrays have been used by six independent groups to study endometriosis [16–21]. However, only Yamagata et al. [20] and Dyson et al. studied purified stromal cells, and only the work of Dyson et al. [21] described methylation and expression differences in normal and endometrial stromal cells of ovarian lesions. In their combined genome-wide methylation and expression analysis in stromal cells of patients with endometriosis versus controls they utilized 450K Methylation Beadchips and expression arrays. Their methylation analyses had a very lenient cutoff, requiring DFM >0.15 with no p-value criterion, which led to a very large set of 9021 DM genes which, as would be expected from such a lenient cutoff, included 96% of the DM CpGs that we have identified using our more stringent criteria.

In agreement with our current findings, the results of Dyson et al. also suggested that genome-wide differences in DNA methylation occur more frequently in the body of the genes as well as in the areas that flank CpG islands. We significantly extended these observations here, showing that DM in endometriosis stromal cells affects specific classes of cis-regulatory elements, and pinpointing genes that pass stringent statistical criteria for DM+DE. Despite their lenient cutoffs for DM, 77 out of 403 genes identified by Dyson et al. as having DM+DE overlapped with our DM+DE genes identified at the CpG level (306 genes) and at the regulatory element level (239 genes), representing 25% and 32% respectively, of the genes in our lists. Although not a complete overlap, given the different profiling platforms used (microarrays in Dyson et al. and RNA-Seq in our study), the definite overlap in DM+DE genes across the two independent case series gratifyingly suggests that endometriosis, while manifesting clinical heterogeneity, in fact shows a degree of epigenetic homogeneity. In addition to the novel aspects discussed above, also new in our work is the demonstration of involvement of both 5mC and 5hmC in the dynamic changes in DNA methylation in endometriosis stromal cells, our fine-mapping of methylation patterns using extensive Bis-Seq, our functional testing of methylation-dependence of candidate genes using the response to 5aza-dC, and our tissue-based validation of one of the biologically interesting DM+DE genes by immunostaining.

In summary, our findings confirm and significantly extend the results of prior studies on epigenetic patterning in the stromal cells of endometriosis, thereby defining a consistent epigenetic signature in endometriosis stromal cells that nominates specific transcriptional and signaling pathways as therapeutic targets for this distressing and difficult to treat gynecological condition.

## Websites and GEO Accession Numbers

### Websites

https://david.ncifcrf.gov/; www.broadinstitute.org/gsea; https://www.ncbi.nlm.nih.gov

### GEO accession numbers

All 450K BeadChips array and RNA-seq files from this study are available from GEO database under accession numbers GSE87810, GSE87809 and GSE87621.

## Supporting Information

S1 Fig Validation of DM region within *JAZF1* gene body. A) Graphical representation of the Illumina Beadchips array methylation data for “index “CpG -cg12988813 at the *JAZF1* gene and showing a gain of DNA methylation in OESC vs. CESC is given. The mean values (horizontal line) and the T-test p-values are indicated. B) Bis-seq confirming hyper-methylation in DMR (R1) overlapping an enhancer region in the body of the gene (amplicon 2) are represented as QUMA plots. The number of the Sanger probes and each individual sample ID are indicated on the top. C) Map of *JAZF1* showing the DMR (R1) and including the “index CpG” corresponding to the CpG given in Fig 2 map as “a”. Chromatin state in NHLF and H1-ESC cell lines (ENCODE project) are color coded as described in the USCS browser.
(TIF)

S2 Fig Validation of DM region at the 3’end of the *BDNF* gene. A) Graphical representation of the Illumina Beadchips array methylation data for “index “CpG—cg051895703 at the *BDNF* gene (top panel) and showing a gain of DNA methylation in OESC versus CESC is given. The mean values (horizontal line) and the T-test p-values are indicated. B) Validation and mapping of the DMR in *BDNF* using bis-seq. Although a single DM CpG was identified by the methylation arrays, bis-seq data validate the DMR and show differential methylation in the contiguous CpG (black rectangle). C) Map of *BDNF* showing hyper-methylation at the 3’ end of the gene. The DMR overlaps a region bearing the chromatin marks of strong transcription (green) and overlapping with the body of the *BDNF*-as transcript. In this region, Illumina BeadChips array coverage is low with only one queried CpG as indicated by the 450K probe track. Chromatin state in NHLF and H1-ESC cell lines (ENCODE project) are color coded as described in the USCS browser.
(TIF)

S3 Fig Validation of the DM region at *TGFBR1* 3’UTR. A) Graphical representation of the Illumina Beadchips array methylation data for “index “CpG -cg13827209 (left) at the *TGFBR1* gene showing gain of DNA methylation in OESC versus CESC. The mean values (horizontal line) and the T-test p-values are indicated. B) Validation and mapping of the DMR in *TGFBR* using bis-seq. Although a single DM CpG was identified by the methylation arrays, bis-seq data validate the DMR and show differential methylation in the contiguous CpGs. The number of the Sanger probes and each individual sample ID are indicated on the top C) Map showing hyper-methylation in the 3’UTR of *TGFBR1*. In this region, Illumina BeadChips array coverage is low with only one queried CpG as indicated by the 450K probe track. Chromatin state in NHLF and H1-ESC cell lines (ENCODE project) are color coded as described in the USCS browser.
(TIF)

S4 Fig A substantial subset of DM genes show correlation between methylation and expression. A) The Venn diagrams shows that methylation correlates with expression in 19% of the DM genes. The XY graphs showing a strong negative correlation between the gene expression and the methylation level at the DMR of *JAZF1* (B) *and DAPK1* (C) genes. The XY graphs showing *BDNF* (D) and *TGFBR1* (E) expression level against the fractional methylation of the “index CpGs” on the Illumina Beadchips array for each gene. Notably, *TGFBR1* and *BDNF* showed a strong correlation between expression and methylation (p = 0.0018, rho correlation coefficient = 0.89 and p = 0.005, rho = 0.9, respectively) but did not pass our DM criteria at FDR < .05 since only 1 of the 450K-queried CpGs in each gene showed strong DM (DFM = 0.4, nominal p-value = 0.002 and DFM = 0.4, nominal p-value = 0.007, respectively).
(TIF)

S5 Fig Validation of the levels of expression of *BDNF/BDNF-as*, *JAZF1/JAZF1-as* genes and analysis of BDNF secretion in OESC vs. CESC. Results of Q-PCR showing reduced expression of *JAZF1* (A) in CESC vs. OESC and over-expression of *BDNF* (B) in OESC vs. CESC cells. The levels of expression are plotted as 2^-^Δ^CT^ values after normalization to the CT values of the housekeeping gene. Normalized expression (2^-ΔCT^ values), mean values (horizontal line) and T-test p-values are indicated. No changes of the levels of the antisense transcripts ware seen for both *JAZF1* and *BDNF* (A, B). C) Graphical representation of the levels of BDNF secreted protein in supernatants of cultured OESC and CESC analyzed by ELISA. Total number of n = 4 independent samples per group using technical triplicates were analyzed and the levels of secreted BDNF were calculated in pg/ml media.
(TIF)

S6 Fig With increasing stringency, there is an enrichment in DM genes showing correlation between methylation and expression. A) Graphs showing the enrichment of genes with correlation between methylation and expression in DM CpGs and segments as a function of the stringency. The ORs become higher with increasing stringency, confirming the robustness of the enrichment. B) The methylation distribution in CpGs with correlation between methylation and expression shows a shift of the usually observed low and high methylation peaks toward the intermediate methylation levels in OESC but not in CESC.
(TIF)

S7 Fig CpGs with correlation between methylation and expression are enriched in enhancers and show element-specific methylation distribution. A) CpGs with correlation between methylation and expression are enriched in enhancers and insulators but depleted in promoter regions. B) Methylation distributions of CpGs with correlation between methylation and expression according to the overlapping regulatory elements. The methylation distributions for CpGs not correlating with expression are, as expected, bimodal in enhancers and insulators but unimodal in promoters. The methylation distributions of CpGs correlating with expression show an enrichment of the intermediate methylation levels in OESC for all the tested regulatory elements. In insulators, the methylation distribution becomes unimodal with only a low methylation peak in both CESC and OESC.
(TIF)

S8 Fig Effects of 5Aza-dC on *ORS2* and *HAND2 DM* and DE genes in CESC and OESC. This figure shows changes in average gene expression levels for ORS2 (A) and HAND2 (B) genes in CESC and OESC under 5Aza-dC treatment for 72 hrs. The levels of expression were normalized to the respective vanish CESC and OESC controls set to 1 for each experimental group.
(TIF)

S1 Table Endometrial and endometriotic samples used for primary cultures preparation and analyzed for DNA methylation and expression. List of endometrial and endometriotic samples used for DNA methylation and expression analysis after primary cell culture preparation are shown. BT ID numbers correspond to the specific sample number in the laboratory tissue bank is given to identify the sample in the corresponding analysis. The sample ID number corresponds to the tissue bank number of the sample in the collaborators lab.
(XLSX)

S2 Table CpGs with differential methylation (DM) in endometriosis stroma cells. This is a complete list of DM loci identified using stringent (A) and lenient (B) criteria for DM between cases and controls. For the stringent criteria, the cut-off was set at FDR<0.05 and absolute difference in fractional methylation of >0.15, and more lenient criteria the nominal p-value<0.05 and at least 2 CpGs in each gene with p-value<0.05 and fractional methylation >0.15 were used. The P-values for the case/control comparison were calculated using Student’s T-tests and false discovery rates were calculated using the Benjamini-Hochberg method. Samples of normal peripheral blood (PBL) and placenta are included in S2B as heterologous tissues for comparison.
(XLSX)

S3 Table Gene-regulatory elements with differential methylation in endometriotic stromal cells. This is a complete list of DM on gene-regulatory elements (See Materials and Methods for definition) identified using stringent (A) and lenient (B) criteria for DM between cases and controls. The stringent criteria are set as a cut-off of FDR<0.05 and absolute difference in averaged fractional methylation >0.10 (or >0.15 when the segment contained a single CpG). The more lenient criteria, are defined at nominal p-value<0.05 and at least 2 segments in a given gene with p-value<0.05 and fractional methylation >0.10 (or >0.15 for single CpG segment). The P-values for the case/control comparison were calculated using Student’s T-tests and false discovery rates were calculated using the Benjamini-Hochberg method.
(XLSX)

S4 Table Gene-ontology (GO) and gene-set (GS) enrichment analysis for DM and DE genes between endometrial and endometriotic stroma cells. The list of the top GO terms enriched in genes identified to be DM by our stringent CpG (A) and segment (B) criteria are given. P-value cut-off <0.05 was used. C) The list of top gene sets showing enrichment of DM regulatory regions identified by our stringent segment criteria and localized in multiple genes including estrogen-responsive genes are given. FDR cut-off <0.005 was used. The list of the top GSEA gene sets and GO terms enriched in genes identified to be DE are shown in (D) and (E), respectively. The same statistical criteria were used as described for DM gene analysis.
(XLSX)

S5 Table List of Differentially methylated and expressed genes in endometriotic stroma. This is a complete list of DM and DE genes in endometriotic stroma identified by overlapping the gene lists of DM genes identified by our stringent (A) and lenient criteria (B) with the gene list created by computational analysis of the CpGs and genomic segments with strong correlation between methylation and expression (cut-off p<0.005 and correlation coefficient>0.7; average FPKM>1 in at least one of the groups) independently of the disease status.
(XLSX)

S6 Table Comparison of methylation levels of DM CpGs correlating with expression and DM CpGs not correlating with expression. (XLSX)

S7 Table Bisulfite PCR primers. (XLSX)

S8 Table Q‐PCR primers. (XLSX)

S9 Table Summary of previous genome-wide methylation studies in endometriosis. (XLSX)
