# Supplementary figures and images for: Epigenetic Alterations Affecting Transcription Factors and Signaling Pathways in Stromal Cells of Endometriosis
Source: PLoS One. 2017 Jan 26;12(1):e0170859. doi: 10.1371/journal.pone.0170859 (PMC5268815; doi:10.1371/journal.pone.0170859)

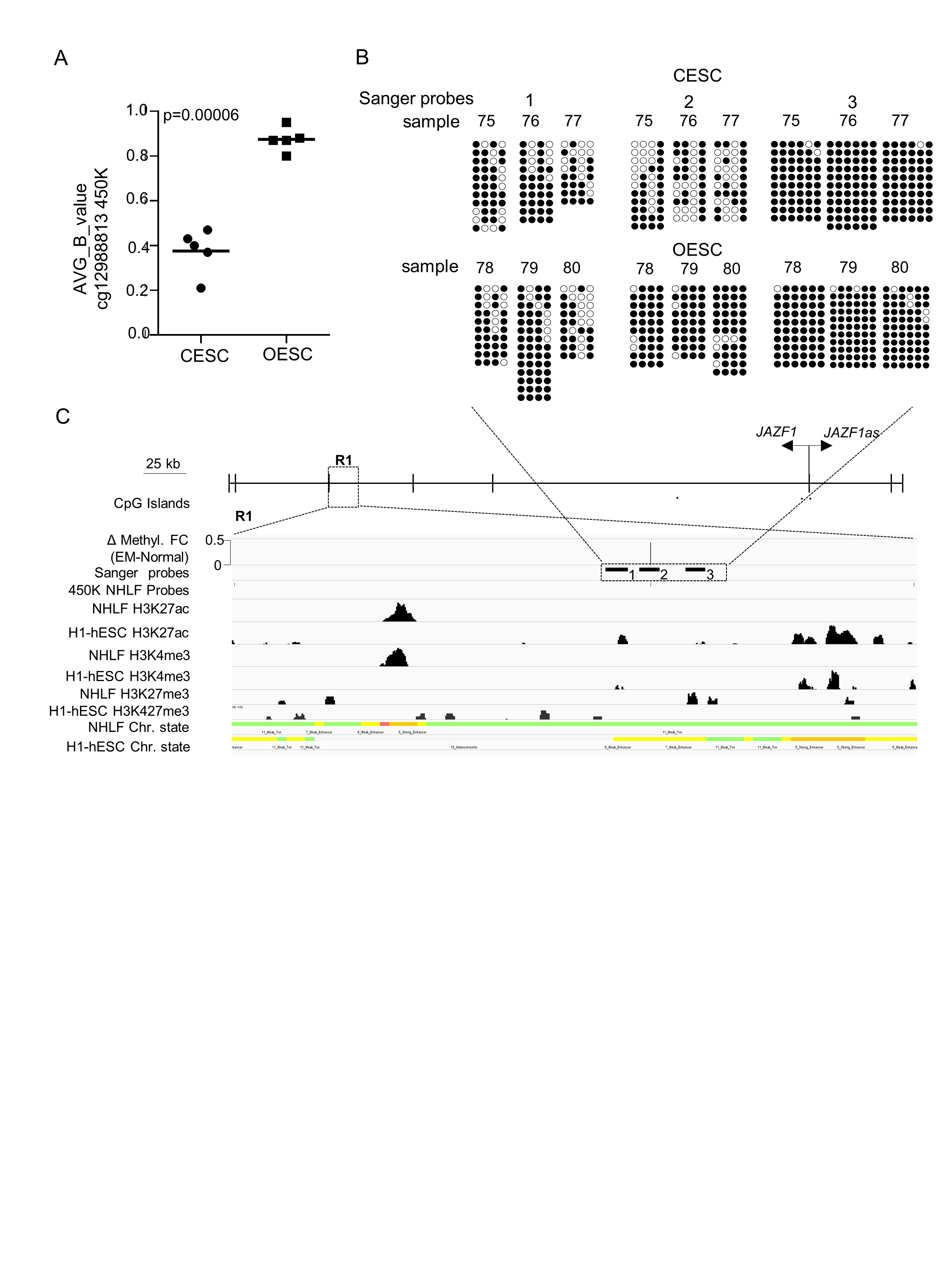

Supplement: S1 Fig — A) Graphical representation of the Illumina Beadchips array methylation data for “index “CpG -cg12988813 at the JAZF1 gene and showing a gain of DNA methylation in OESC vs. CESC is given. The mean values (horizontal line) and the T-test p-values are indicated. B) Bis-seq confirming hyper-methylation in DMR (R1) overlapping an enhancer region in the body of the gene (amplicon 2) are represented as QUMA plots. The number of the Sanger probes and each individual sample ID are indicated on the top. C) Map of JAZF1 showing the DMR (R1) and including the “index CpG” corresponding to the CpG given in Fig 2 map as “a”. Chromatin state in NHLF and H1-ESC cell lines (ENCODE project) are color coded as described in the USCS browser. (TIF) [file pone.0170859.s001.tif]

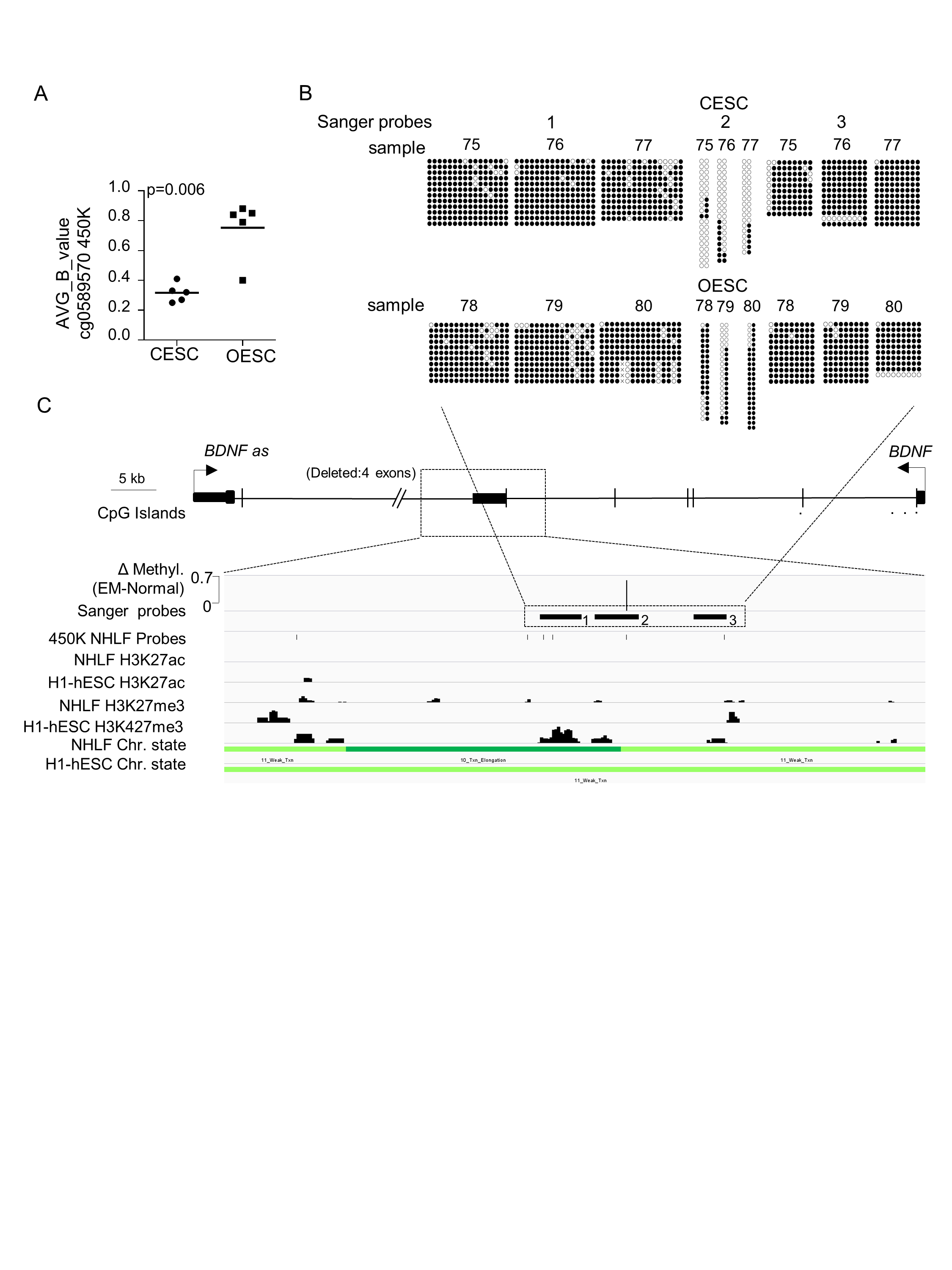

Supplement: S2 Fig — A) Graphical representation of the Illumina Beadchips array methylation data for “index “CpG—cg051895703 at the BDNF gene (top panel) and showing a gain of DNA methylation in OESC versus CESC is given. The mean values (horizontal line) and the T-test p-values are indicated. B) Validation and mapping of the DMR in BDNF using bis-seq. Although a single DM CpG was identified by the methylation arrays, bis-seq data validate the DMR and show differential methylation in the contiguous CpG (black rectangle). C) Map of BDNF showing hyper-methylation at the 3’ end of the gene. The DMR overlaps a region bearing the chromatin marks of strong transcription (green) and overlapping with the body of the BDNF-as transcript. In this region, Illumina BeadChips array coverage is low with only one queried CpG as indicated by the 450K probe track. Chromatin state in NHLF and H1-ESC cell lines (ENCODE project) are color coded as described in the USCS browser. (TIF) [file pone.0170859.s002.tif]

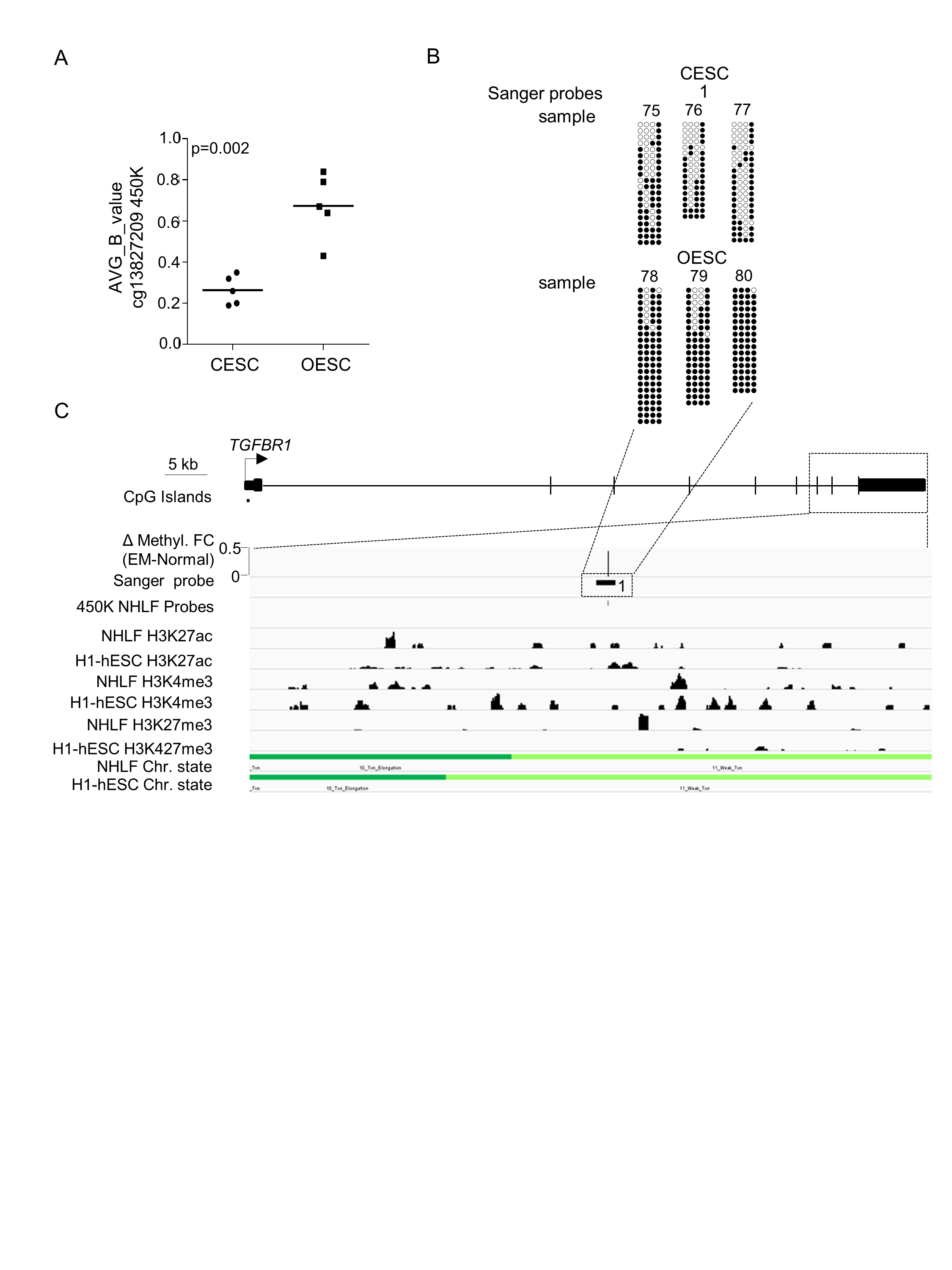

Supplement: S3 Fig — A) Graphical representation of the Illumina Beadchips array methylation data for “index “CpG -cg13827209 (left) at the TGFBR1 gene showing gain of DNA methylation in OESC versus CESC. The mean values (horizontal line) and the T-test p-values are indicated. B) Validation and mapping of the DMR in TGFBR using bis-seq. Although a single DM CpG was identified by the methylation arrays, bis-seq data validate the DMR and show differential methylation in the contiguous CpGs. The number of the Sanger probes and each individual sample ID are indicated on the top C) Map showing hyper-methylation in the 3’UTR of TGFBR1. In this region, Illumina BeadChips array coverage is low with only one queried CpG as indicated by the 450K probe track. Chromatin state in NHLF and H1-ESC cell lines (ENCODE project) are color coded as described in the USCS browser. (TIF) [file pone.0170859.s003.tif]

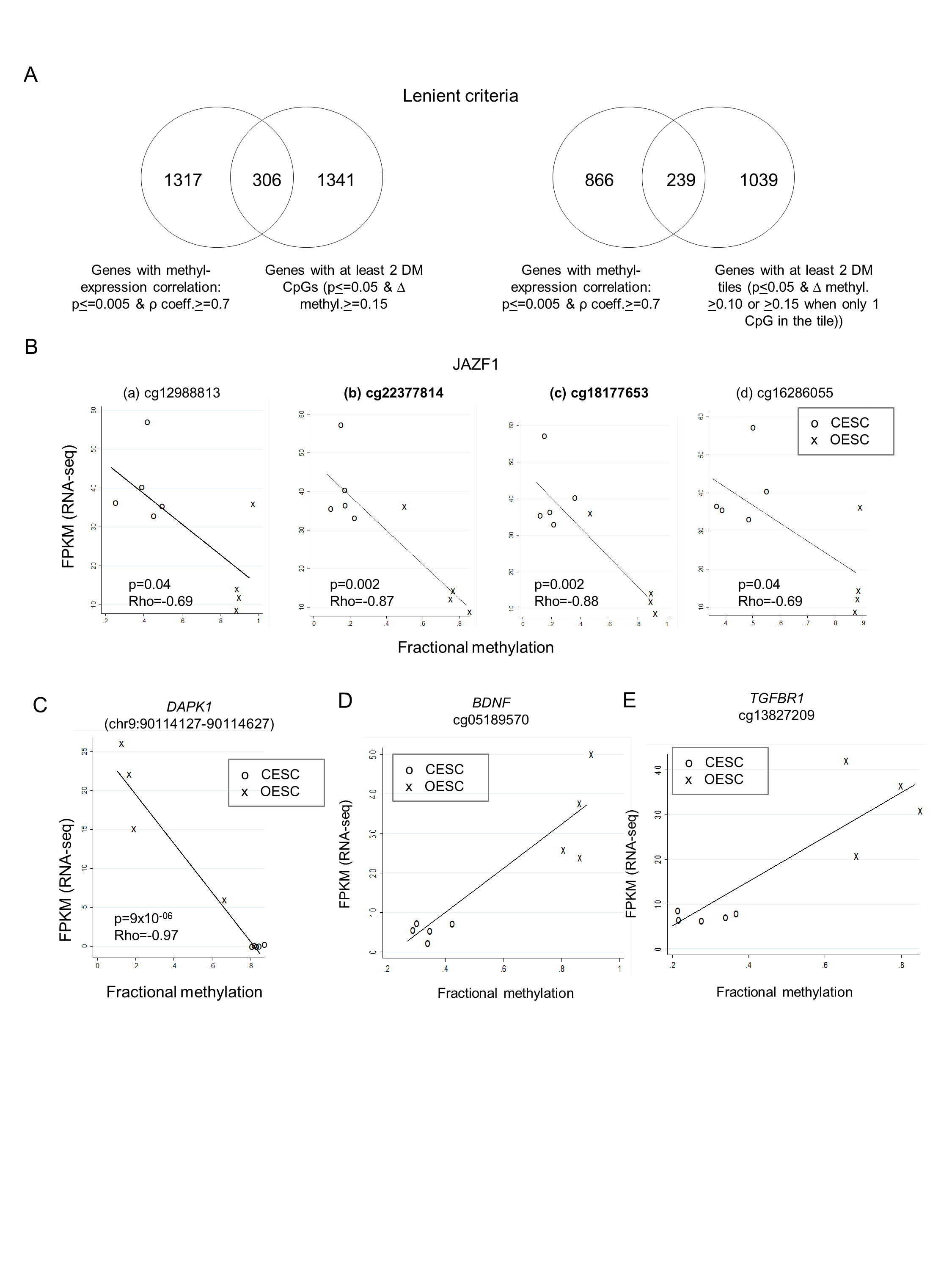

Supplement: S4 Fig — A) The Venn diagrams shows that methylation correlates with expression in 19% of the DM genes. The XY graphs showing a strong negative correlation between the gene expression and the methylation level at the DMR of JAZF1 (B) and DAPK1 (C) genes. The XY graphs showing BDNF (D) and TGFBR1 (E) expression level against the fractional methylation of the “index CpGs” on the Illumina Beadchips array for each gene. Notably, TGFBR1 and BDNF showed a strong correlation between expression and methylation (p = 0.0018, rho correlation coefficient = 0.89 and p = 0.005, rho = 0.9, respectively) but did not pass our DM criteria at FDR < .05 since only 1 of the 450K-queried CpGs in each gene showed strong DM (DFM = 0.4, nominal p-value = 0.002 and DFM = 0.4, nominal p-value = 0.007, respectively). (TIF) [file pone.0170859.s004.tif]

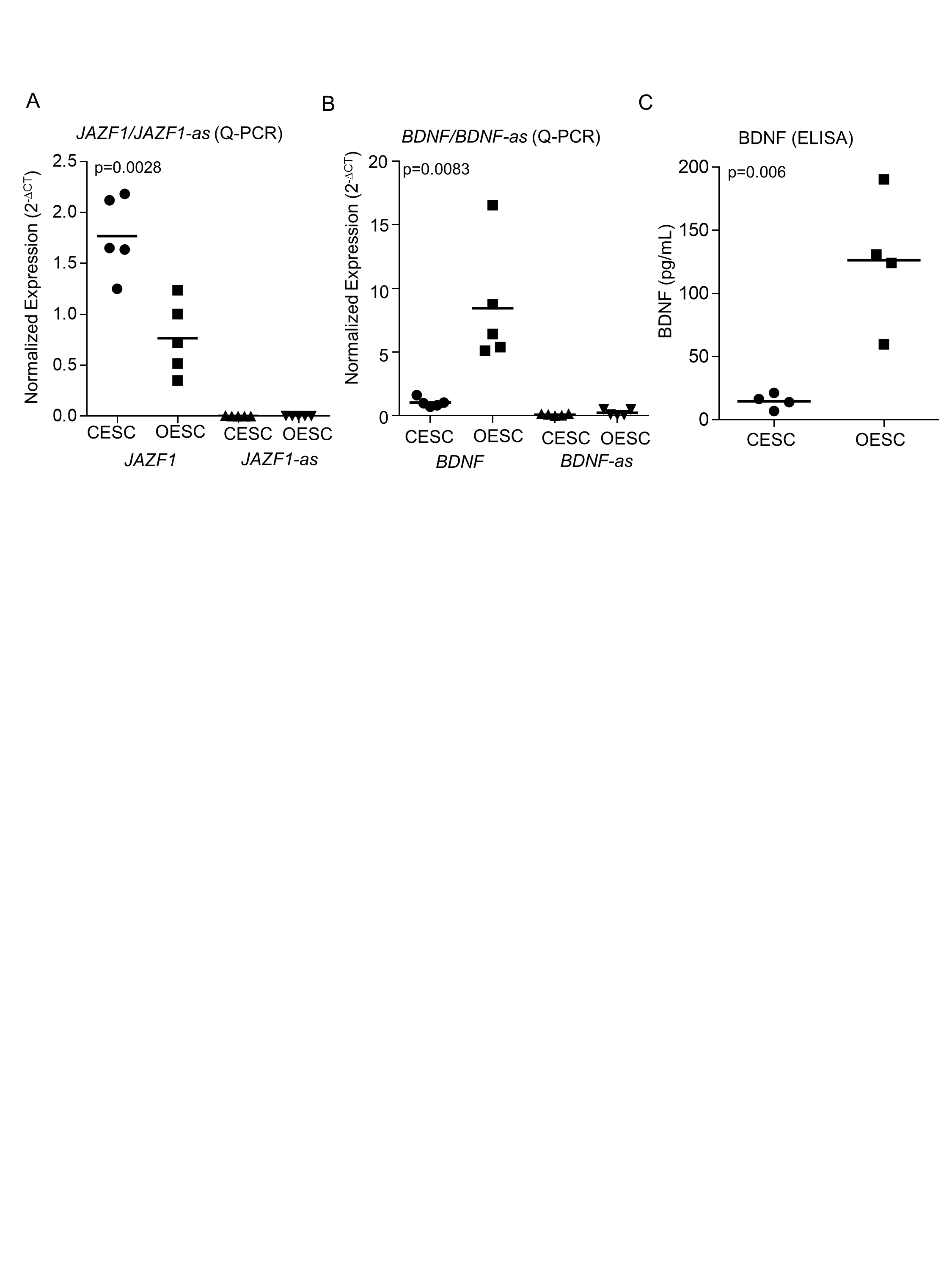

Supplement: S5 Fig — Results of Q-PCR showing reduced expression of JAZF1 (A) in CESC vs. OESC and over-expression of BDNF (B) in OESC vs. CESC cells. The levels of expression are plotted as 2-ΔCT values after normalization to the CT values of the housekeeping gene. Normalized expression (2-ΔCT values), mean values (horizontal line) and T-test p-values are indicated. No changes of the levels of the antisense transcripts ware seen for both JAZF1 and BDNF (A, B). C) Graphical representation of the levels of BDNF secreted protein in supernatants of cultured OESC and CESC analyzed by ELISA. Total number of n = 4 independent samples per group using technical triplicates were analyzed and the levels of secreted BDNF were calculated in pg/ml media. (TIF) [file pone.0170859.s005.tif]

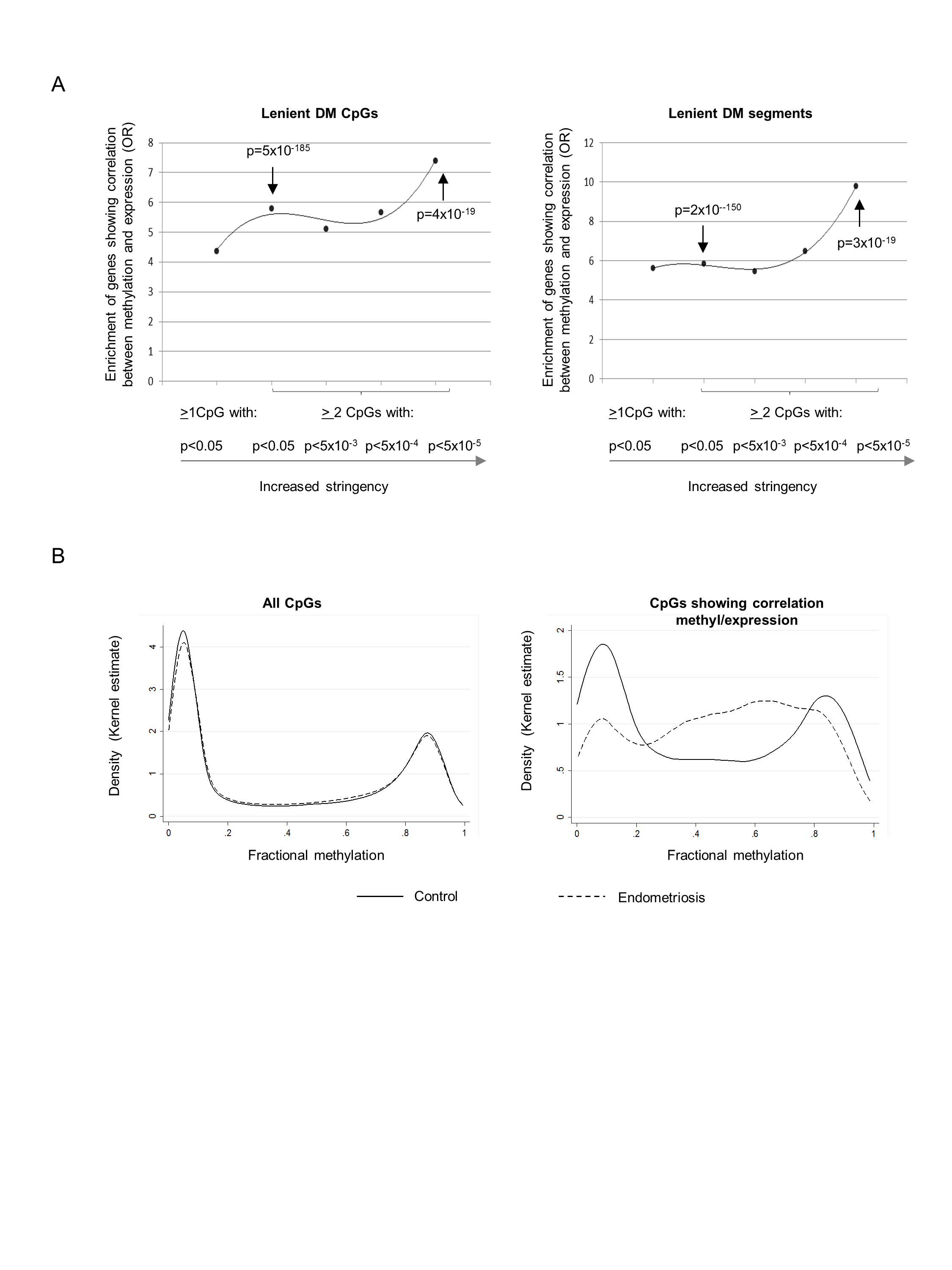

Supplement: S6 Fig — A) Graphs showing the enrichment of genes with correlation between methylation and expression in DM CpGs and segments as a function of the stringency. The ORs become higher with increasing stringency, confirming the robustness of the enrichment. B) The methylation distribution in CpGs with correlation between methylation and expression shows a shift of the usually observed low and high methylation peaks toward the intermediate methylation levels in OESC but not in CESC. (TIF) [file pone.0170859.s006.tif]

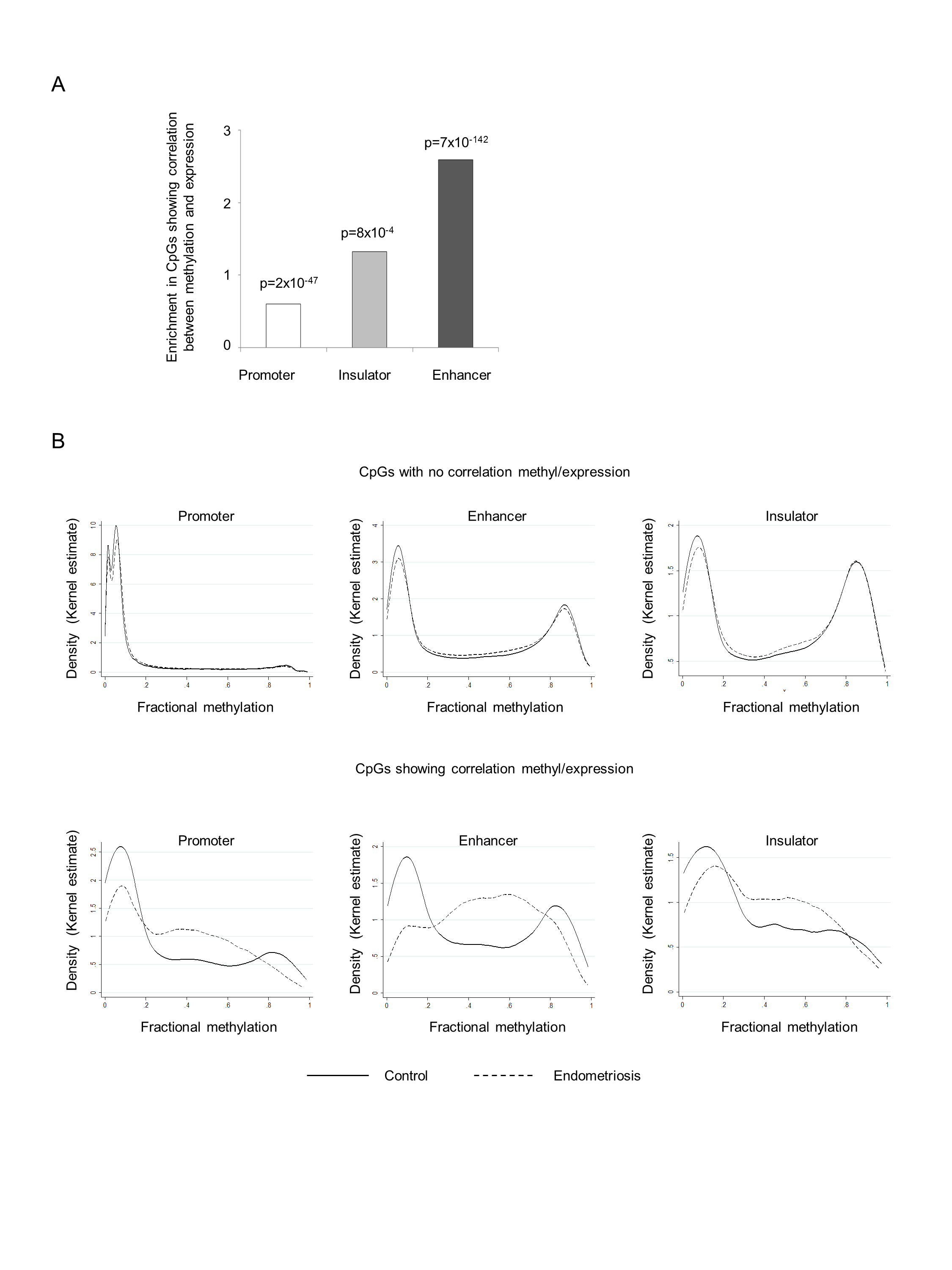

Supplement: S7 Fig — A) CpGs with correlation between methylation and expression are enriched in enhancers and insulators but depleted in promoter regions. B) Methylation distributions of CpGs with correlation between methylation and expression according to the overlapping regulatory elements. The methylation distributions for CpGs not correlating with expression are, as expected, bimodal in enhancers and insulators but unimodal in promoters. The methylation distributions of CpGs correlating with expression show an enrichment of the intermediate methylation levels in OESC for all the tested regulatory elements. In insulators, the methylation distribution becomes unimodal with only a low methylation peak in both CESC and OESC. (TIF) [file pone.0170859.s007.tif]

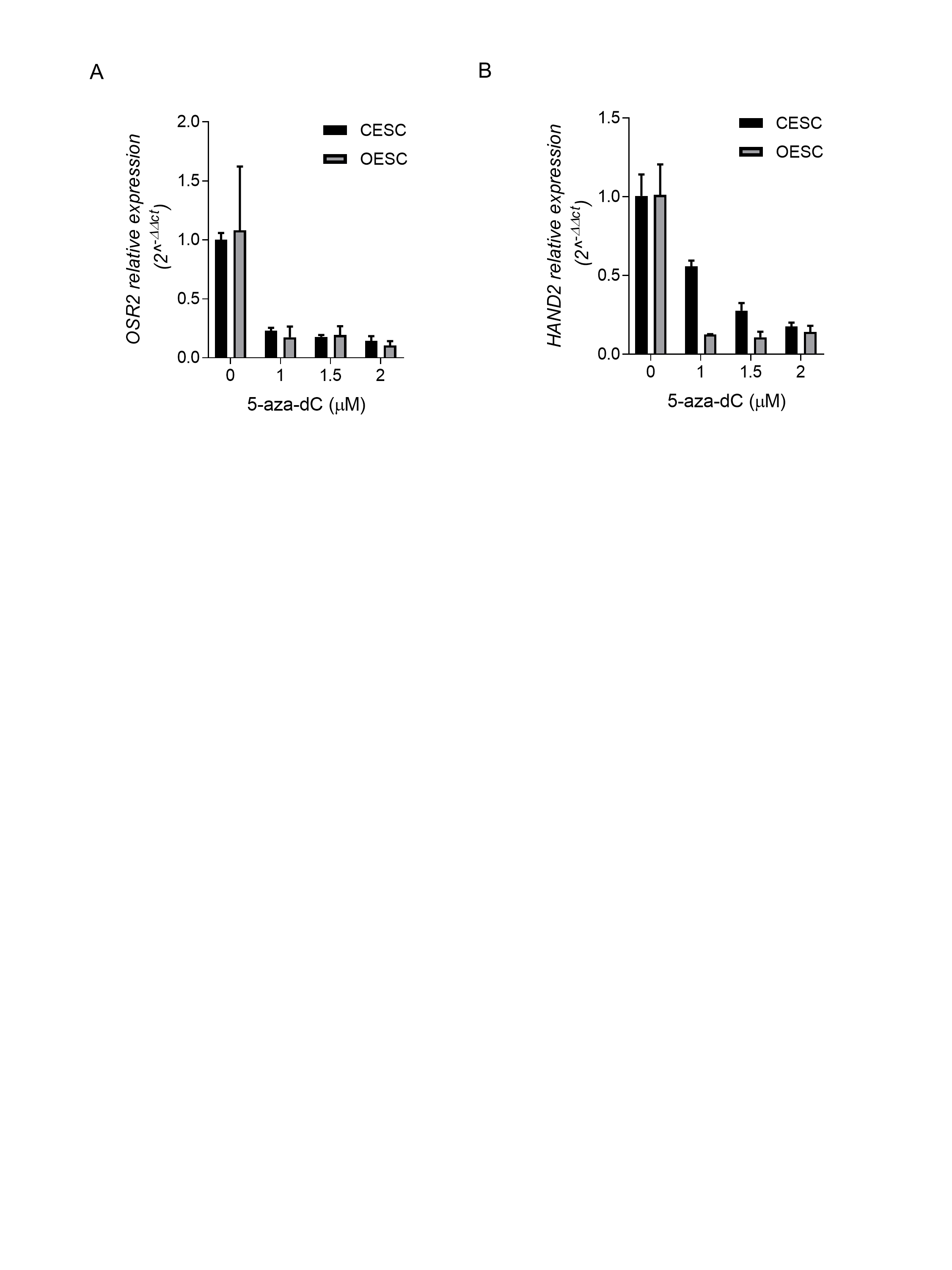

Supplement: S8 Fig — This figure shows changes in average gene expression levels for ORS2 (A) and HAND2 (B) genes in CESC and OESC under 5Aza-dC treatment for 72 hrs. The levels of expression were normalized to the respective vanish CESC and OESC controls set to 1 for each experimental group. (TIF) [file pone.0170859.s008.tif]
